# Coinage Metal Complexes of the Carbenic Tautomer of A Conjugated Mesomeric Betaine Akin to Nitron †

**DOI:** 10.3390/molecules22071133

**Published:** 2017-07-07

**Authors:** Charlotte Thie, Clemens Bruhn, Michael Leibold, Ulrich Siemeling

**Affiliations:** Institute of Chemistry, University of Kassel, Heinrich-Plett-Straße 40, 34132 Kassel, Germany; Charlotte_Thie@gmx.de (C.T.); c.bruhn@uni-kassel.de (C.B.); m.leibold@uni-kassel.de (M.L.)

**Keywords:** conjugated mesomeric betaine, coinage metals, copper, crystal structure, gold, *N*-heterocyclic carbene, silver, 1,2,4-triazol-5-ylidene

## Abstract

This study was motivated by our recent observation that the analytical reagent Nitron (**2**) is an “instant carbene”, whose reaction with coinage metal salts MX afforded complexes of its carbenic tautomer 1,4-diphenyl-3-phenylamino-1,2,4-triazol-5-ylidene (**2**′). Our aim was to establish an alkyl homologue of **2** in order to achieve a carbenic tautomer of higher donicity. For this purpose 1-*tert*-butyl-4-methyl-1,2,4-triazol-4-ium-3-*tert*-butylaminide (**6**) was synthesized. Its reactions with MX afforded complexes of the carbenic tautomer 1-*tert*-butyl-3-*tert*-butylamino-4-methyl-1,2,4-triazol-5-ylidene (**6**′). With a stoichiometric ratio of 1:1 complexes of the type [MX(**6**′)] were obtained. A ratio of 2:1 furnished complexes of the type [MX(**6**′)_2_] or [M(**6**′)_2_]X. **6**′ is a better σ-donor and less electrophilic than **2**′ according to NMR spectroscopic data of **6**H[BF_4_] and **6**′ = Se, respectively, and IR spectroscopic data of [RhCl(**6**′)(CO)_2_] confirm that its net electron donor capacity is superior to that of **2**′. A comparison of the complexes of **2**′ and **6**′ reveals two pronounced structural differences. [CuX(**6**′)_2_] (X = Cl, Br) exhibit more acute C–Cu–C bond angles than [CuX(**2**′)_2_]. In contrast to [CuCl(**2**′)], [CuCl(**6**′)] aggregates through Cu···Cu contacts of ca. 2.87 Å, compatible with cuprophilic interactions. These differences may be explained by the complementary steric requirements of the *t*-Bu and the Me substituent of **6**′.

## 1. Introduction

**Figure 1:**
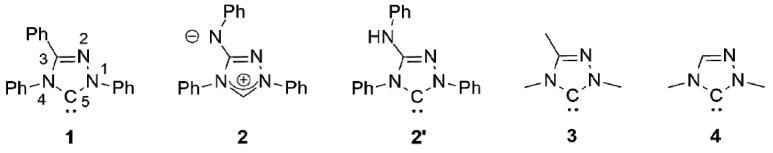
The “Enders carbene” (**1**), Nitron (**2**) and its carbenic tautomer (**2**′), the methyl homologue (**3**) of **1** and 1,4-dimethyl-1,2,4-triazol-5-ylidene (**4**).

The chemistry of coinage metal complexes containing *N*-heterocyclic carbene (NHC) ligands has been expanding rapidly due to the relevance of such compounds in catalysis, materials science, and in particular for pharmaceutical applications [1,2,3,4,5,6,7,8,9,10,11,12,13,14,15,16,17,18,19,20,21]. 1,2,4-Triazol-5-ylidenes have been neglected in this context. This is illustrated by the fact that the first coinage metal complexes of the iconic “Enders carbene” (1,3,4-triphenyl-1,2,4,triazol-5-ylidene, **1**; Figure 1), [Ag(**1**)_2_]X (X = BF_4_, PF_6_) and [AgCl(**1**)], were published only recently [22,23] and is furthermore also evident from the very limited number of systematic studies concerning such complexes published to date [24,25,26,27,28,29,30].

We recently described the use of the analytical reagent Nitron (**2**; Figure 1) as an “instant carbene” [31,32,33], whose reaction with coinage metal salts MX in dipolar aprotic solvents afforded complexes of its carbenic tautomer 1,4-diphenyl-3-phenylamino-1,2,4-triazol-5-ylidene (**2**′; Figure 1) under mild conditions [33]. With a stoichiometric ratio of 1:1 complexes of the type [MX(**2**′)] were obtained; a stoichiometric ratio of 2:1 furnished complexes of the type [MX(**2**′)_2_] or [M(**2**′)_2_]X.

Nitron belongs to the class of conjugated mesomeric betaines (CMBs) [34]. Related work on other five-membered CMBs in equilibrium with their tautomeric NHCs was published by the groups of César and Lavigne [35,36,37], Braunstein and Danopoulos [38,39,40], Ganter [41] and Schmidt [42,43,44,45,46,47,48,49], who also provided the first review of this burgeoning field [50]. We demonstrated that the electronic properties of **2**′ are very similar to those of the “Enders carbene” (**1**) [31,32,33], as is reflected by their essentially identical Tolman Electronic Parameter (TEP) values [51] as well as by the very similar ^77^Se NMR chemical shift values (∆δ = 4 ppm) determined for their corresponding selenone derivatives. In our present work we describe a homologue containing *N*-alkyl substituents instead of the *N*-phenyl substituents present in **2**′. A comparison of the HOMO energies and singlet-triplet gaps of **1** and its methyl homologue 1,3,4-trimethyl-1,2,4-triazol-5-ylidene (**3**; Figure 1) indicates that alkyl homologues of **1** are comparatively more nucleophilic and less electrophilic [52]. Although experimental evidence in support of these computational results is not available for the particular pair **1** and **3**, the data obtained for corresponding derivatives of **1** and 1,4-dimethyl-1,2,4-triazol-5-ylidene (**4**; Figure 1) clearly show that the alkyl-substituted NHC is less electrophilic (δ(^77^Se): 20 vs. 110 ppm for the respective selenone derivative [53] **4** = Se [54] and **1** = Se [33]) and has a higher net electron donor capacity than **1** (TEP value according to the linear regression given by Dröge and Glorius [55]: 2054 [56] vs. 2057 cm^−1^ [57] for **4** and **1**, respectively). Similar effects may plausibly be expected for alkyl homologues of **2**′.

## 2. Results and Discussion

### 2.1. Compound Synthesis and Spectroscopic Characterization

**Scheme 1:**
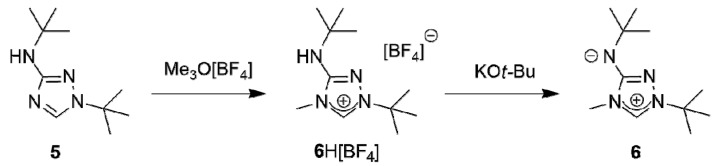
Synthesis of the new CMB **6** from 1-*tert*-butyl-3-*tert*-butylamino-1,2,4-triazole (**5**).

A suitable starting point for our work was provided by the recent report that the commercially available 3-amino-1,2,4-triazole is readily di-*tert*-butylated under mild conditions on a multigram scale by a mixture of *tert*-butanol and 65% aqueous perchloric acid to furnish 1-*tert*-butyl-3-*tert*-butylamino-1,2,4-triazol-4-ium perchlorate, whose subsequent reaction with dilute aqueous sodium hydroxide cleanly affords 1-*tert*-butyl-3-*tert*-butylamino-1,2,4-triazole (**5**) [58]. The methylation of this compound with trimethyloxonium tetrafluoroborate in dichloromethane occurred exclusively at *N*4 (Scheme 1); 1-*tert*-butyl-3-*tert*-butylamino-4-methyl-1,2,4-triazol-4-ium tetrafluoroborate (**6**H[BF_4_]) was obtained almost quantitatively. The methylation also worked well with methyl iodide. However, this route was abandoned, because purification of **6**HI was tedious and the iodide anion was found to cause problems in the following steps due to its tenacity and redox-active nature.

**Scheme 2:**
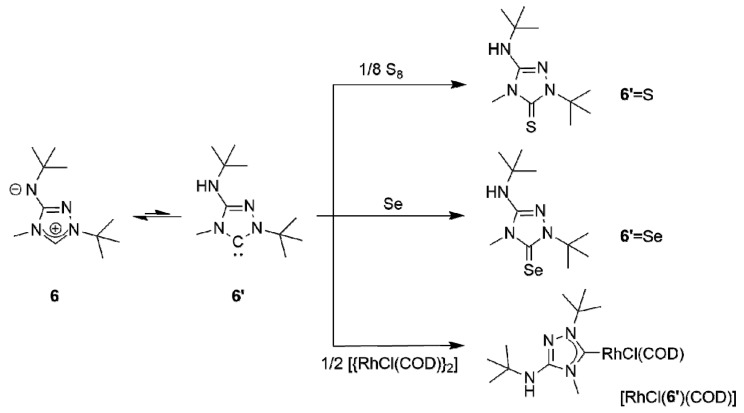
Reactions of CMB **6** (shown in postulated equilibrium with its carbenic tautomer **6**′) with typical carbene-trapping reagents.

The subsequent reaction of **6**H[BF_4_] with potassium *tert*-butoxide in a mixture of THF and toluene afforded the new CMB 1-*tert*-butyl-4-methyl-1,2,4-triazol-4-ium-3-*tert*-butylaminide (**6**) in excellent yield. Although a ^1^H-NMR spectroscopic analysis gave no indication for the presence of the carbenic tautomer 1-*tert*-butyl-3-*tert*-butylamino-4-methyl-1,2,4-triazol-5-ylidene (**6**′) in solution, this CMB reacted readily with typical carbene-trapping reagents, *viz.* elemental sulfur and selenium as well as [{Rh(µ-Cl)(COD)}_2_] (COD = cycloocta-1,5-diene) (Scheme 2). The role of the carbenic tautomer **6**′ in these reactions is supported by results of DFT calculations (BP86/def2-SVP, for details see the corresponding section of the Supplementary Materials) which reveal that in the gas phase **6**′ is only slightly less stable than 6 (∆*G*^0^ = 4.7 kcal mol^−1^ for the process **6** → **6**′; for comparison, ∆*G*^0^ = 5.7 kcal mol^−1^ for the process **2** → **2**′ [31]).

The corresponding heterourea-type derivatives **6**′ = E (E = S, Se) as well as the rhodium(I) NHC complex [RhCl(**6**′)(COD)] were isolated in good to excellent yields. Due to the hindered rotation around the Rh–C_carbene_ bond, the CH and CH_2_ units of the COD ligand each give rise to four signals in the ^13^C-NMR spectrum. This behavior is completely analogous to that of Nitron (**2**) [31,33]. [RhCl(**6**′)(COD)] was converted to the dicarbonyl complex [RhCl(**6**′)(CO)_2_] by stirring a solution of the diolefin chelate complex in dichloromethane under an atmosphere of carbon monoxide. As expected, our spectroscopic data show that **6**′ is less electrophilic than **2**′ ((δ(^77^Se): 89 vs. 106 ppm for **6**′ = Se and **2**′ = Se [33], respectively, in CDCl_3_) and also has a comparatively higher net electron donor capacity (TEP value according to the linear regression given by Dröge and Glorius [55]: 2054 vs. 2057 cm^−1^ [31] for **6**′ and **2**′, respectively). This is further corroborated by the ^1^*J*(^1^H,^13^C) coupling constant observed for the triazolium C–H unit of **6**H[BF_4_], which is smaller than that found for **2**H[BF_4_] (224 vs. 229 Hz [33]). It has been pointed out that such coupling constants correlate inversely with the σ-donor strengths of the respective carbenes, since the σ-donor strength decreases with increasing s-character of the σ-orbital at the divalent carbon atom [59,60] and large ^1^*J*(^1^H,^13^C) coupling constants reflect high s-character of the carbon valence orbital involved in the C–H bond [61].

**Figure 2:**
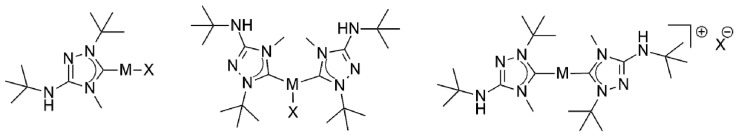
Plausible structures of the coinage metal complexes of the present study. (**Left**) “1:1 complexes” (structurally characterized examples: [MCl(**6**′)], M = Cu, Au). Note that “1:1 complexes” obtained from silver(I) halides may isomerize to [M(**6**′)_2_][MX_2_]. (**Middle**) Undissociated “2:1 complexes” (structurally characterized examples: [AgBr(**6**′)_2_] and [CuX(**6**′)_2_], X = Cl, Br). (**Right**) Dissociated ionic “2:1 complexes” (structurally characterized examples: [Ag(**6**′)_2_]X, X = Br, OTf).

We subsequently addressed the synthesis of coinage metal complexes of **6**′ from the CMB **6** and coinage metal salts MX, in analogy to our recent work with Nitron (**2**) in this context (Figure 2) [33]. Similar to our results obtained with **2**, the synthesis of copper(I) complexes of the type [CuX(**6**′)] and [CuX(**6**′)_2_] (X = Cl, Br, I) was very straightforward. The use of equimolar amounts of CuX and **6** afforded the expected “1:1 complexes” [CuCl(**6**′)], [CuBr(**6**′)] and [CuI(**6**′)] in isolated yields of 80, 92 and 98%, respectively. The differences in yield mildly correlate with the solubility differences of these complexes. In comparison to the analogous complexes of **2**′, the solubility of the 1:1 copper(I) complexes of **6**′ in solvents of low to moderate polarity such as, for example, benzene, chloroform or dichloromethane, is higher, which facilitated their NMR spectroscopic characterization. The C_carbene_ atom gives rise to a diagnostic low-field ^13^C-NMR signal located at δ = 171.5 ppm for the chlorido complex [CuCl(**6**′)] and at δ = 172.8 and 176.1 ppm, respectively, for the bromido and iodido complex (CDCl_3_ solution). The same slight increase of the chemical shift of the C_carbene_ signal (Cl < Br < I) was observed before for the analogous complexes of **2**′ [33], which, however, were investigated in DMSO-*d*_6_ solution for solubility reasons. This trend is well known and correlates with the σ-donor capacity of the halide ligand, which increases in the same order [62]. The signal due to the quaternary carbon atom of the heterocyclic ring, which bears the exocyclic amino group, is located at δ ≈ 151 ppm in each case. This value is essentially identical to that found for the analogous complexes of **2**′ in DMSO-*d*_6_ solution [33]. The NH*t*-Bu substituent causes a characteristic NH signal in the ^1^H-NMR spectrum at δ = 3.94, 3.84 and 3.63 ppm for the chlorido, bromido and iodido complex, respectively. The corresponding signal of the analogous complexes of **2**′ was observed at much lower field (δ ≈ 9.0 ppm), which can be ascribed mainly to the fact that DMSO-*d*_6_ was used as solvent, causing the engagement of the NHPh moiety in hydrogen bonding.

The use of two equivalents of **6** furnished the “2:1 complexes” [CuX(**6**′)_2_] (X = Cl, Br, I) in essentially quantitative yields. The NMR spectroscopic characterization of these compounds was severely hampered by their very poor solubility in common organic solvents, which was significantly lower than that of the analogous complexes of **2**′. ^1^H and ^13^C{^1^H} NMR spectra of satisfactory quality could be recorded only at elevated temperatures (typically at 70 °C) in DMSO-*d*_6_. In the case of [CuX(**2**′)_2_] two C_carbene_ signals were observed in the ^13^C-NMR spectra; likewise, the ^1^H-NMR spectra exhibited two NH signals. This was ascribed to the partial dissociation of the X^–^ ligand in DMSO-*d*_6_ solution at room temperature, giving rise to [Cu(**2**′)_2_]^+^. In contrast, only a single C_carbene_ signal is present in the ^13^C-NMR spectra of [CuX(**6**′)_2_]. In the same vein, the ^1^H-NMR spectra contain only a single NH signal. This suggests that a single species is dominantly present in DMSO-*d*_6_ solution at 70 °C, which could be either the undissociated or the dissociated ionic form. In view of the elevated temperature used and the higher donicity of **6**′ in comparison to **2**′, we surmise that the dissociated form [Cu(**6**′)_2_]^+^ is more likely here.

In addition to the reactions of **6** with the copper halides CuX, we also addressed the behavior of this new CMB towards AgCl and AgBr, since Nitron (**2**) was previously found to furnish the corresponding 1:1 and 2:1 Silver(I) complexes of its carbenic tautomer **2**′ in good to excellent yield, despite the insoluble nature of these silver halides [33] Indeed, the corresponding complexes of **6**′ were obtained analogously. These complexes show fairly good solubility in solvents of low to moderate polarity, which was beneficial for NMR spectroscopic studies. No ^1^*J*(^13^C,^109/107^Ag) couplings were observed, pointing to a rapid exchange of the carbene ligands on the NMR time scale, as is frequently described for silver(I) complexes of five-membered ring NHCs [62]. In the case of complexes obtained from a silver halide AgX and an NHC in a stoichiometric ratio of 1:1 such fluxional behavior is known to give rise to mixtures of [AgX(NHC)] and [Ag(NHC)_2_][AgX_2_] in solution [1]. Such mixtures may be avoided by using less nucleophilic anions Y^−^, which favor the formation of [Ag(NHC)_2_]Y at the expense of [AgY(NHC)]. Therefore, silver triflate was allowed to react with two equivalents of **6**, which cleanly afforded [Ag(**6**′)_2_](OTf) in 93% isolated yield. The NMR spectroscopic behavior of this compound is very similar to that of [Ag(**2**′)_2_](OTf) [33]. In particular, the C_carbene_ atoms give rise to a pair of doublets (δ = 173.3 ppm in CDCl_3_) with nicely resolved couplings between carbon and silver of ^1^*J*(^13^C,^109^Ag) ≈ 213 Hz and ^1^*J*(^13^C,^1^0^7^Ag) ≈ 190 Hz, indicating that the silver-carbon bond is not labile [62,63,64]. Finally, we also investigated the reaction of **6** with [AuCl(THT)] (THT = tetrahydrothiophene). In our previous work with Nitron (**2**), we found that complexes of the composition AuCl(**2**′)*_n_* (*n* = 1, 2) were easily obtained by this method as air-stable solids in excellent yield [33]. In contrast, [AuCl(**6**′)] is prone to unspecific decomposition. This occurred slowly in the solid state (even in the absence of light) and was fairly rapid in solution, where the formation of elemental gold was observed. This behavior hampered the NMR spectroscopic characterization of this compound. Similar stability problems were not encountered when two equivalents of **6** were used, which afforded a complex of the composition AuCl(**6**′)_2_ in74% isolated yield. This complex is plausibly formulated as [Au(**6**′)_2_]Cl, since gold(I) chloride is known to form 2:1 complexes of the type [Au(NHC)_2_]Cl with standard NHCs [1,65,66]. However, to the best of our knowledge only two structurally characterized example are known to date with 1,2,4-triazol-5-ylidene ligands in this context [67]. In view of the comparatively weak, more phosphine-like donicity of 1,2,4-triazol-5-ylidene ligands a three-coordinate structure cannot be ruled out with certainty, therefore, similar to structures determined for a variety of phosphine complexes of the type [AuCl(PR_3_)_2_] [68,69,70,71].

### 2.2. Structural Characterization

**Figure 3:**
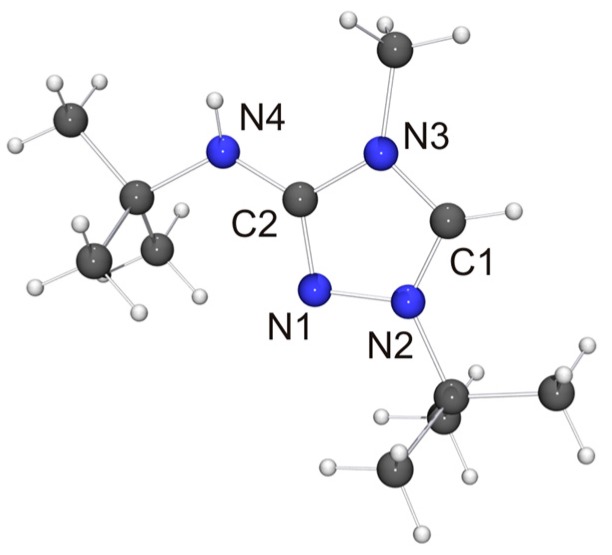
Molecular structure of the cation of **6**H[BF_4_] in the crystal. Selected bond lengths [Å] and angles [°]: C1–N2 1.310(1), C1–N3 1.340(1), C2–N1 1.320(1), C2–N3 1.380(1), C2–N4 1.351(1), N1–N2 1.383(1); N2–C1–N3 107.74(9).

We have been able to perform single-crystal X-ray diffraction studies for most of the new compounds described in the previous section. The molecular structure of the cation of 1-*tert*-butyl-3-*tert*-butylamino-4-methyl-1,2,4-triazol-4-ium tetrafluoroborate (**6**H[BF_4_]) is shown in Figure 3.

Not unexpectedly, the bond lengths and angles of **6**H^+^ are very similar to those of the cation of **2**HCl [32] and (**2**H)_2_[Co(NCS)_4_] [72] (i.e., protonated Nitron), which is the only other structurally characterized 3-amino-substituted 1,2,4-triazolium species reported to date. The anion of **6**H[BF_4_] is located close to the exocyclic amino unit. One of the F atoms exhibits a weak contact to this unit (NH···F 2.44 Å, not shown in Figure 3), which is ca. 8% below the sum of the van der Waals radii of F and H (2.66 Å) [73]. We note that the NH···F distance is longer than the NH···Cl distance (2.36 Å) between the amino unit and the hydrogen-bonded chloride anion of **2**HCl [32], which is not surprising in view of the inferior hydrogen bond acceptor ability of tetrafluoroborate [74].

**Figure 4:**
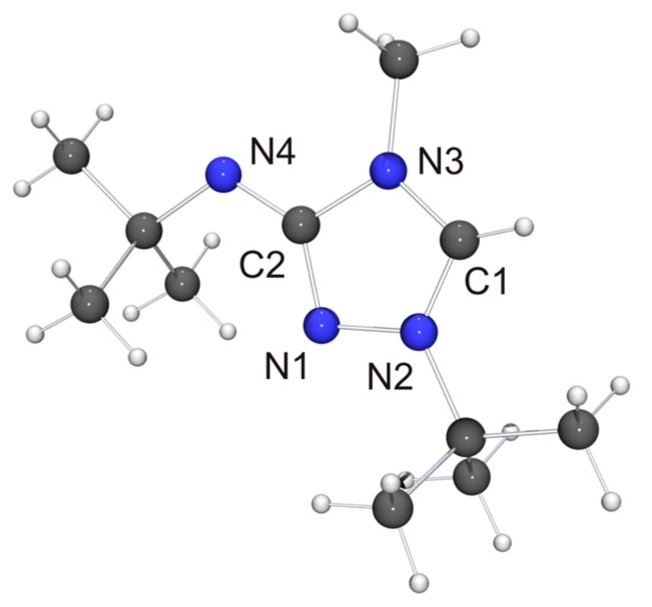
Molecular structure of **6** in the crystal. Selected bond lengths [Å] and angles [°]: C1–N2 1.315(2), C1–N3 1.339(2), C2–N1 1.364(2), C2–N3 1.420(2), C2–N4 1.308(2), N1–N2 1.383(2); N2–C1–N3 107.46(13).

The molecular structure of the new CMB **6** is shown in Figure 4. It is in good agreement with the computed structure (see Table S4 of the Supplementary Materials). The only closely related 1,2,4-triazole-based compound that has been structurally characterized to date is **2**·½EtOH [75], in which case, however, the precision and definition of the structure determination was adversely affected by decomposition and solvent disorder. Nevertheless, it is obvious that **2** and **6** share structural features which are distinctly different from those of the respective protonated species. First, the bond between the exocyclic nitrogen atom and the carbon atom of the heterocyclic ring is shorter in the case of the CMB (approximately 1.31 vs. 1.35 Å for **6** and **6**H[BF4], respectively; approximately 1.32 vs. 1.36 Å for **2** [75] and **2**Cl·MeOH [32], respectively), which suggests a significant degree of π-delocalization of the anionic charge of the aminide unit into the heterocyclic ring. This structural effect is also seen in related imidazole-based CMBs [35,38,39]. Second, both CMBs exhibit N_2_CH···NR contacts between the 1,2,4-triazolium and aminide moieties of neighboring molecules (not shown in Figure 4). The corresponding hydrogen–nitrogen distances are ca. 2.08 and 2.35 Å for **2** and ca. 2.48 Å for **6**, which is considerably shorter in each case than the sum of the van der Waals radii of H and N (2.86 Å) [73] and compatible with weak hydrogen bonds [76,77].

**Figure 5:**
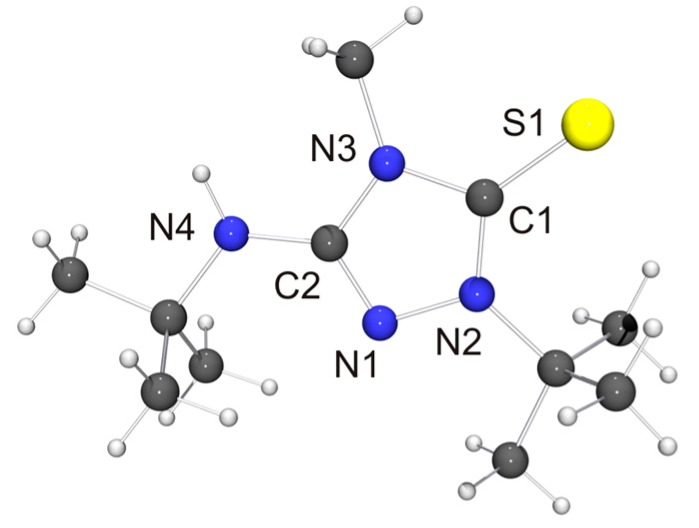
Molecular structure of the cation of thione **6**′ = S in the crystal. Selected bond lengths [Å] and angles [°]: C1–N2 1.335(3), C1–N3 1.388(3), C1–S1 1.689(3), C2–N1 1.301(3), C2–N3 1.365(4), C2–N4 1.374(3), N1–N2 1.399(3); N2–C1–N3 104.3(2).

The molecular structure of the thione derivative **6**′ = S is shown in Figure 5. NH···S contacts (2.73 Å) are present between neighboring molecules (not shown in Figure 5), which are ca. 12% shorter than the sum of the van der Waals radii of S and H (3.09 Å) [73] and compatible with weak hydrogen bonds [78]. Intermolecular NH···S interactions have been observed before for thiourea-type derivatives [79]. The bond lengths and angles of the 1,2,4-triazoline-5-thione unit of **6**′ = S are very similar to those reported for simple alkyl-substituted examples such as 1,4-dimethyl-1,2,4-triazole-5-thione (**4** = S) [80]. They are also very similar to those determined for the only example so far which bears an additional NHR unit in the 3-position [81].

**Figure 6:**
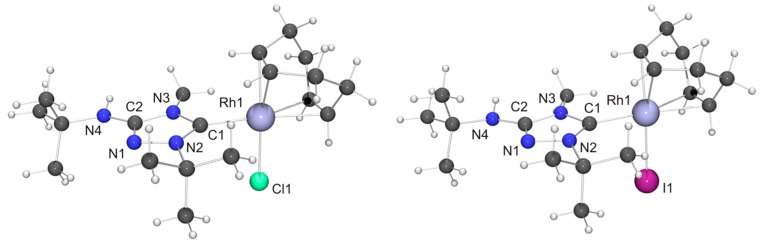
(**Left**) Molecular structure of [RhCl(**6**′)(COD)] in the crystal. Only one of the two independent molecules present in the asymmetric unit is shown. Selected bond lengths [Å] and angles [°]: C1–Rh1 2.054(5), C1–N2 1.325(6), C1–N3 1.369(6), C2–N1 1.314(6), C2–N3 1.367(6), C2–N4 1.360(6), C12–Rh1 2.220(4), C13–Rh1 2.205(5), C16–Rh1 2.146(4), C17–Rh1 2.107(4) 2, N1–N2 1.392(5), Cl1–Rh1 2.3885(12); N2–C1–N3 103.1(4), C1–Rh1–Cl1 87.91(13); (**Right**) Molecular structure of [RhI(**6**′)(COD)] in the crystal. Only one of the two independent molecules present in the asymmetric unit is shown. Selected bond lengths [Å] and angles [°]: C1–Rh1 2.050(4), C1–N2 1.332(5), C1–N3 1.372(5), C2–N1 1.296(6), C2–N3 1.382(5), C2–N4 1.371(6), C12–Rh1 2.143(4), C13–Rh1 2.101(4), C16–Rh1 2.217(4), C17–Rh1 2.211(4), N1–N2 1.405(5), I1–Rh1 2.6738(4); N2–C1–N3 103.3(3), C1–Rh1–I1 88.71(12).

We now come to the precious metal complexes of **6**′. The molecular structure of [RhCl(**6**′)(COD)] is shown in Figure 6, together with that of the iodido homologue [RhI(**6**′)(COD)], which we obtained by serendipity in a preliminary experiment, where we used **6**HI to generate **6**, which was contaminated with residual iodide (*vide supra*). Two independent molecules are present in the asymmetric unit in each case, whose bond parameters differ only marginally. In the case of the chlorido complex, NH···Cl contacts of ca. 2.76 and 2.81 Å are present between neighboring molecules (not shown in Figure 6). These distances are ca. 8% shorter than the sum of the van der Waals radii of Cl and H (3.02 Å) [73] and fall in the intermediate range of M–Cl···H–N hydrogen bonds according to an established classification [82]. Likewise, neighboring molecules of the iodido homologue are associated through NH···I contacts (ca. 2.90 Å; not shown in Figure 6), which are ca. 10% shorter than the sum of the van der Waals radii of I and H (3.24 Å) [73]. In both compounds the Rh^I^ atom is in the distorted square-planar coordination environment typical of tetracoordinate d^8^ transition metal complexes and exhibits a bond distance of ca. 2.05 Å to the C_carbene_ atom. The Rh–C_COD_ bond lengths are significantly longer for the carbon atoms *trans* to the NHC ligand than for those *trans* to the halido ligand (average value ca. 2.21 vs. 2.13 Å in each case), which can be ascribed to the comparatively high *trans* influence [83] of NHC ligands [84,85,86,87]. These structural features compare well with those of other complexes of the type [RhX(NHC)(COD)] with 1,2,4-triazol-5-ylidene ligands [88,89,90], including also the Nitron-derived homologue [RhCl(**2**′)(COD)] [31].

**Figure 7:**
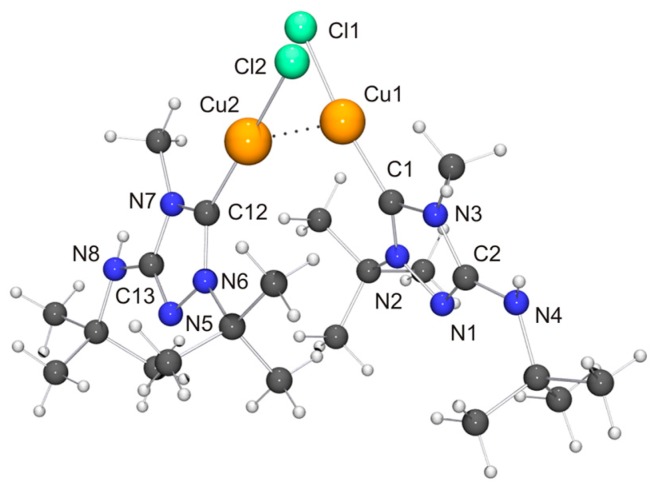
Molecular structure and aggregation of [CuCl(**6**′)]·CH_2_Cl_2_ in the crystal. The solvent molecule has been omitted for clarity. The Cu···Cu contact compatible with a cuprophilic interaction is indicated by a dotted line. Selected bond lengths [Å] and angles [°] for molecule 1: C1–Cu1 1.886(3), C1–N2 1.324(4), C1–N3 1.379(4), C2–N1 1.306(5), C2–N3 1.374(4), C2–N4 1.362(4), N1–N2 1.398(4), Cu1–Cl1 2.1251(9), Cu1–Cu2 2.8687(6); N2–C1–N3 102.9(3), C1–Cu1–Cl1 171.47(11).

**Figure 8:**
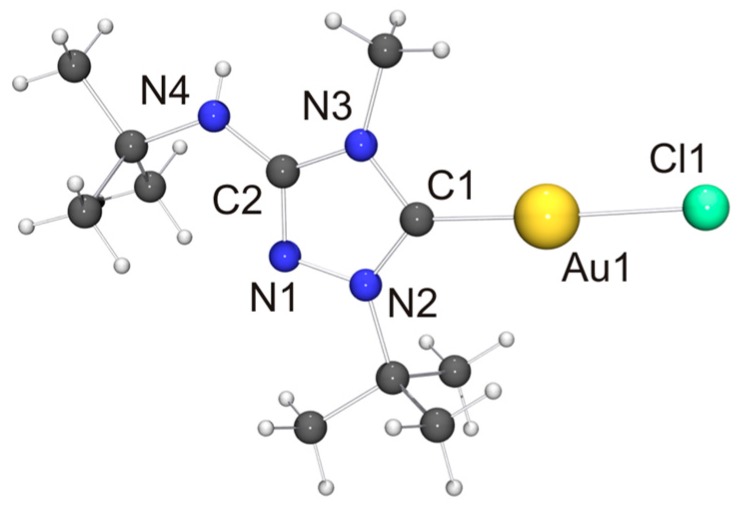
Molecular structure and aggregation of [AuCl(**6**′)] in the crystal. Selected bond lengths [Å] and angles [°]: C1–Au1 1.975(7), C1–N2 1.342(8), C1–N3 1.370(9), C2–N1 1.311(8), C2–N3 1.363(8), C2–N4 1.359(9), N1–N2 1.362(7), Au1–Cl1 2.2874(16); N2–C1–N3 103.3(6), C1–Au1–Cl1 176.3(2).

In comparison to our previous work with Nitron (**2**), it was more difficult to obtain crystals of coinage metal complexes of the carbenic tautomer of **6** suitable for single-crystal X-ray diffraction studies. Among the “1:1 complexes”, we have succeeded only in two cases, *viz.* [CuCl(**6**′)] (Figure 7) and [AuCl(**6**′)] (Figure 8).

Two independent molecules with very similar bond parameters are present in the asymmetric unit of [CuCl(**6**′)], which are aggregated through a Cu···Cu contact of 2.8687(6) Å. This distance is in the range considered to be typical of so-called cuprophilic interactions [91,92,93,94,95,96,97], which in a recent theoretical study were compared to hydrogen bonds [98]. The metallophilic aggregation of 1,2,4-triazol-5-ylidene coinage metal complexes of the type [MX(NHC)] (M = Cu, Ag, Au; X = F, Cl, Br, I) has been addressed computationally in a recent systematic study, which focused exemplarily on NHC = 1,3,4-trimethyl-1,2,4-triazol-5-ylidene (**3**) [99]. It was found that the [MX(**3**)] molecules form head-to-tail dimers, which exhibit metal···metal distances in the range between 3.04 (for the dimer of [AgI(**3**)]) and 3.64 Å (for the dimer of [AgF(**3**)]). A good agreement was achieved between the computed gas phase and the experimentally determined solid state structures of the corresponding gold(I) chlorido complex, which constituted the only complex in this series which according to the authors had been structurally characterized by single-crystal X-ray diffraction [100]. In particular, the difference between the calculated and the experimentally determined Au···Au distance (3.19 vs. 3.33 Å) was judged to be within acceptable limits. The Cu···Cu distance calculated for the dimer of [CuCl(**3**)] was 3.34 Å, which is much longer than the value of 2.8687(6) Å determined by us for [CuCl(**6**′)]. Furthermore, [CuCl(**6**′)] does not form head-to-tail dimers in the solid state, as is evident from the Cl–Cu–Cu–Cl and C–Cu–Cu–Cl torsion angles of ca. 70°. Consequently, the structure may best be described as a crossed dimer. In this staggered arrangement steric repulsions are lower than in an eclipsed orientation and consequently a closer approach of the two metal atoms is possible. The two C–Cu–Cl units of a dimer are each quasi-linear, the copper bond angle being 171.47(11) and 174.39(10)°, respectively. This deviation from linearity reflects the intermetallic contact present in the dimer. For comparison, the corresponding complex derived from Nitron (**2**), [CuCl(**2**′)], does not exhibit a similar aggregation and has an essentially linear C–Cu–Cl unit with a bond angle of 177.88(10)° [33]. The bond lengths of this unit are almost identical for [CuCl(**2**′)] and [CuCl(**6**′)]. We finally note that in the case of the latter complex neighboring dimers are connected through NH···Cl contacts (not shown in Figure 7) of ca. 2.48 and 2.65 Å, which respectively fall in the short and intermediate range of M–Cl···H–N hydrogen bonds according to the previously mentioned classification [82].

In view of the Cu···Cu contact found for [CuCl(**6**′)], we expected a similar intermetallic contact for [AuCl(**6**′)]. Indeed, neighboring molecules exhibit an Au···Au distance of 3.6120(9) Å (not shown in Figure 8), compatible with weak aurophilic interactions [101,102,103,104]. The Cl–Au–Au–Cl and C–Au–Au–Cl torsion angles are ca. 150 and 154°, respectively, indicating an approximate head-to-tail arrangement of the molecules in the dimeric aggregate. This structural motif is not uncommon for complexes of the type [AuX(NHC)] containing functionalized NHC ligands [105], including [AuCl(**2**′)] [33]. The almost linear C–Au–Cl unit, which exhibits a gold bond angle of 176.3(2)°, further indicates that the intermetallic interaction is very weak. The bond lengths of this unit as well as those of the 1,2,4-triazol-5-ylidene unit are nearly identical for [AuCl(**2**′)] and [AuCl(**6**′)] and also very similar to those of the very few other structurally characterized complexes of the type [AuCl(NHC)] containing 1,4-dialkyl-substituted 1,2,4-triazol-5-ylidene ligands known to date [24,28].

**Figure 9:**
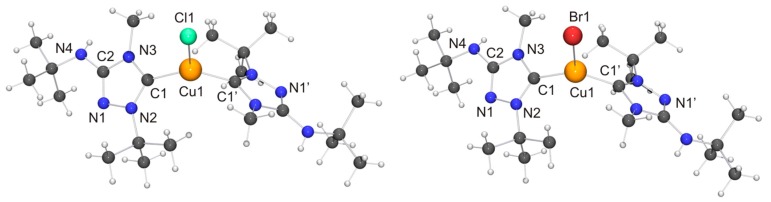
(**Left**) Molecular structure of [CuCl(**6**′)_2_] in the crystal. Selected bond lengths [Å] and angles [°]: C1–Cu1 1.940(6), C1–N2 1.348(7), C1–N3 1.366(7), C2–N1 1.308(8), C2–N3 1.372(7), C2–N4 1.357(8), N1–N2 1.405(7), Cu1–Cl1 2.336(2); N2–C1–N3 102.6(5), C1–Cu1–C1 129.5(3), C1–Cu1–Cl1 115.25(16); (**Right**) Molecular structure of [CuBr(**6**′)_2_] in the crystal. Selected bond lengths [Å] and angles [°]:C1–Cu1 1.914(5), C1–N2 1.322(6), C1–N3 1.353(6), C2–N1 1.287(6), C2–N3 1.355(6), C2–N4 1.358(7), N1–N2 1.384(6), Cu1–Br1 2.4324(10); N2–C1–N3 103.0(4), C1–Cu1–C1 132.7(3), C1–Cu1–Br1 113.64(15).

**Figure 10:**
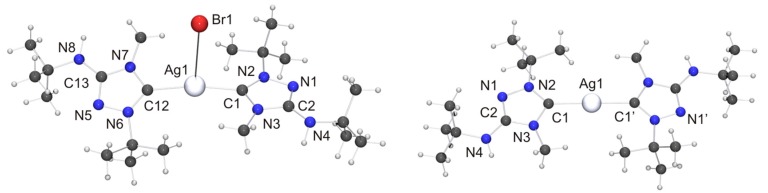
(**Left**) Molecular structure of [AgBr(**6**′)_2_]·toluene in the crystal. The solvent molecule has been omitted for clarity. Selected bond lengths [Å] and angles [°]: C1–Ag1 2.107(7), C1–N2 1.327(9), C1–N3 1.364(9), C2–N1 1.302(9), C2–N3 1.375(9), C2–N4 1.356(9), C12–Ag1 2.121(7), C12–N6 1.312(9), C12–N7 1.360(9), C13–N5 1.312(9), C13–N7 1.360(9), C13–N8 1.363(9), N1–N2 1.401(8), N5–N6 1.401(8), Ag1–Br1 3.0938(9); N2–C1–N3 103.0(6), C1–Ag1–C12 169.2(2), C1–Ag1–Br1 89.4(2), C12–Ag1–Br1 101.4(2); (**Right**) Molecular structure of the cation of [Ag(**6**′)_2_]Br in the crystal. Selected bond lengths [Å] and angles [°]: C1–Ag1 2.098(3), C1–N2 1.335(4), C1–N3 1.369(4), C2–N1 1.315(5), C2–N3 1.379(4), C2–N4 1.355(5), N1–N2 1.388(4); N2–C1–N3 102.7(3), C1–Ag1–C1 178.8(2).

Among the “2:1 complexes”, we have been able to perform single-crystal X-ray diffraction studies for [CuX(**6**′)_2_] (X = Cl, Br), [Ag(**6**′)_2_](OTf) and two isomers of the complex obtained with AgBr, *viz.* the dissociated ionic form [Ag(**6**′)_2_]Br and the undissociated form [AgBr(**6**′)_2_]. The molecular structures of the copper(I) complexes are shown in Figure 9. Those of [AgBr(**6**′)_2_] and the cation of the silver(I) complex [Ag(**6**′)_2_]Br are shown in Figure 10.

Both copper(I) complexes exhibit crystallographically imposed molecular *C*_2_ symmetry. Their copper–carbon bond lengths are indistinguishable within experimental error from those of the corresponding complexes obtained with Nitron (**2**), which constitute the only structurally characterized 1,2,4-triazol-5-ylidene complexes of the type [CuX(NHC)_2_] known to date. Their respective copper–halogen bond is slightly, but significantly, shorter than that of the corresponding complex containing **2**′ (∆*d* ≈ 5 and 6 pm for X = Cl and Br, respectively). Another difference between the complexes of **6**′ and their homologues containing **2**′ concerns their C–Cu–C bond angles. This is more acute for the complexes of **6**′, only slightly for X = Br (≈3°) and much more pronounced for X = Cl (≈13°). This points to a less bulky nature of **6**′ vs. **2**′ in this context, due to the fact that the heterocyclic core of the latter contains two identical *N*-substituents of moderate steric bulk (Ph), while the former has instead two quite different *N*-substituents (Me, *t*-Bu), whose arrangement in the *C*_2_ symmetric species [CuX(**6**′)_2_] reflects their complementary steric requirements. In the crystal structure of [CuCl(**6**′)_2_] neighboring molecules are associated through NH···Cl contacts of ca. 2.79 Å (not shown in Figure 9). This value is ca. 8% shorter than the sum of the van der Waals radii of Cl and H (3.02 Å) [73] and falls in the intermediate range of M–Cl···H–N hydrogen bonds according to the classification mentioned above [82]. [CuBr(**6**′)_2_] exhibits analogous NH···Br contacts of ca. 2.90 Å (not shown in Figure 9), which is ca. 5% shorter than the sum of the van der Waals radii of Br and H (3.06 Å) [73].

We now turn our attention to the Silver(I) complexes obtained with two equivalents of **6**. For complexes formed from imidazole-based NHCs with AgBr which exhibit a stoichiometric ratio of 2:1 of both components two principal structure types are known, *viz.* dicoordinate [Ag(NHC)_2_]Br [106] and tricoordinate [Ag(NHC)_2_Br] [107,108,109]. The dicoordinate structure type is routinely observed with less nucleophilic anions such as, for example, nitrate, perchlorate, triflate, tetrafluoroborate or hexafluorophosphate [1]. Structurally characterized homologues containing 1,2,4-triazol-5-ylidene ligands have not been reported to date for AgBr. However, we note that the di- and tricoordinate type was respectively found for AgCl [25] and AgI [26], indicating that both structure types may be possible with AgBr. Indeed, this turned out to be the case. When toluene was used as solvent for recrystallization of the “2:1 complex” obtained from **6** and AgBr, the undissociated isomer [AgBr(**6**′)_2_] was obtained as toluene solvate (Figure 10, left). In contrast, the more polar solvent acetone favored the dissociated ionic isomer [Ag(**6**′)_2_]Br (Figure 10, right). The Ag–C bond lengths of [AgBr(**6**′)_2_] (average value 2.11 Å) are essentially identical with those of tricoordinate complexes of the type [Ag(NHC)_2_Br] containing imidazole-based NHC ligands [107,108,109]. In contrast, the Ag–Br distance is much longer (3.0938(9) vs. ca. 2.90 Å) and the C–Ag–C bond angle significantly larger (169.2(2)° vs. ca. 160°) than in the tricoordinate imidazole-based NHC complexes. This indicates that the bromido ligand of [AgBr(**6**′)_2_] is coordinated only weakly and can undergo facile dissociation. The loose coordination of the bromido ligand to the Ag^I^ atom coincides with two NH···Br contacts of ca. 2.55 Å to neighboring molecules (not shown in Figure 10), which are ca. 17% shorter than the sum of the van der Waals radii of H and Br (3.06 Å) [73] and compatible with moderately strong hydrogen bonds [82]. The cation of the dissociated isomer [Ag(**6**′)_2_]Br exhibits non-crystallographic *C_i_* symmetry. The Ag–C bond lengths are very similar to those of the undissociated isomer. The best planes of the two heterocyclic rings adopt an almost coplanar orientation, forming a dihedral angle of only 7.3° (the corresponding angle of [AgBr(**6**′)_2_]·toluene is 31.0°). This is made possible by the complementary steric requirements of the methyl and *tert*-butyl substituents, which are facing each other in the cation. We note that even in the case of the sterically uncongested complex [Ag(**4**)_2_](NO_3_) the best planes of the two 1,2-dimethyl-1,2,4-triazol-5-ylidene rings exhibit a similar twist of ca. 11° with respect to one another [110]. The bromide anion is weakly hydrogen-bonded to one of the exocyclic amino groups, as is indicated by the NH···Br contact of 2.77 Å (not shown in Figure 10), which is ca. 9% shorter than the sum of the van der Waals radii of H and Br (3.06 Å) [73]. The silver bond parameters (Ag–C ca. 2.10 Å, C–Ag–C ca. 179°) of [Ag(**6**′)_2_]Br compare well with those of the only example so far of an imidazole-based complex which belongs to the dicoordinate structure type [Ag(NHC)_2_]Br [106]. They are also in good agreement with the values reported for 1,2,4-triazol-5-ylidene complexes of the dicoordinate type [Ag(NHC)_2_]X containing less nucleophilic anions (for example, X = NO_3_ [110], ClO_4_ [27], OTf [89]), including the recently reported complexes of the “Enders carbene”, [Ag(**1**)_2_]X (X = BF_4_, PF_6_) [22] as well as our Nitron-derived examples [Ag(**2**′)_2_][BF_4_] and [Ag(**2**′)_2_](OTf) [33]. The latter compound is the homologue of [Ag(**6**′)_2_](OTf) synthesized and structurally characterized in this work. Unfortunately, the quality of the crystal structure determination of [Ag(**6**′)_2_](OTf) was affected by symmetry problems. [Ag(**6**′)_2_]^+^ appears to be centrosymmetric, while the triflate anion is disordered by interchanging the S and C positions. A calculation in *P*1 revealed the complete order of the anion, but regrettably compromised the *R* values and further quality parameters. Consequently, a discussion is meaningful only for the fundamental features. Not unexpectedly, these are similar to those of [Ag(**2**′)_2_](OTf) [33]. The cation exhibits an essentially linear dicoordinate Ag^I^ atom. The average Ag–C_carbene_ bond length is ca. 2.06 Å. The best planes of the two five-membered rings form a dihedral angle of ca. 9°, which means that the two 1,2,4-triazol-5-ylidene moieties are almost coplanar (similar also to [Ag(**6**′)_2_]Br). The anion of [Ag(**6**′)_2_](OTf) appears to be weakly hydrogen-bonded to the exocyclic amino unit of one of the 1,2,4-triazol-5-ylidene ligands, as is suggested by an NH···O contact of ca. 2.51 Å, which is ca. 7% shorter than the sum of the van der Waals radii of H and O (2.70 Å) [73].

## 3. Experimental Section

### 3.1. General

All reactions were performed in an inert atmosphere (argon or dinitrogen) by using standard Schlenk techniques or a conventional glovebox. Gold and silver compounds were handled with exclusion of light. 1-*tert*-Butyl-3-*tert*-butylamino-1,2,4-triazole (**5**) [58] and [AuCl(THT)] (THT = tetrahydrothiophene) [111] were synthesized by adapted versions of published procedures. A Hettich ROTINA 46 RS centrifuge suitable for Schlenk tubes was used to separate precipitates, which could not easily be removed by filtration. ^1^H- and ^13^C-NMR spectra were recorded with Varian MR-400 and Varian NMRS-500 spectrometers operating at 400 and 500 MHz, respectively, for ^1^H. ^77^Se NMR spectra were recorded with a Varian NMRS-500 spectrometer. Neat dimethylselenide was used as external standard (δ = 4 ppm) [112]. High-resolution (HR) ESI mass spectra were obtained with a micrOTOF time-of-flight mass spectrometer (Bruker Daltonics, Bremen, Germany) using an Apollo^TM^ “ion funnel” ESI source. Mass calibration was performed immediately prior to the measurement with ESI Tune Mix Standard (Agilent, Waldbronn, Germany). FTIR spectroscopy was performed in situ with a Mettler-Toledo ReactIR 15 instrument equipped with a SiComp probe. Elemental analyses were carried out with a HEKAtech Euro EA-CHNS elemental analyzer at the Institute of Chemistry, University of Kassel, Kassel, Germany.

### 3.2. X-ray Crystallography

For each data collection a single crystal was mounted on a micro mount and all geometric and intensity data were taken from this sample. Diffraction experiments were performed on either a Stoe IPDS2 diffractometer equipped with a 2-circle goniometer and an area detector or a Stoe StadiVari diffractometer equipped with a Dectris Pilatus 200K detector. The data sets were corrected for absorption (by integration), Lorentz and polarization effects. Data collections were carried out using Mo*K*_α_ radiation (*λ* = 0.71073 Å) except for **6**, for which Cu*K*_𝛼_ radiation (*λ* = 1.54186 Å) was used. The structures were solved by direct methods (SIR 2008) [113] and refined using alternating cycles of least-squares refinements against *F*^2^ (SHELXL2014/7) [114]. C-bonded H atoms were included to the models in calculated positions. N-bonded H atoms were found in the difference Fourier list and included to the model with a fixed distance of 1.0 Å. All H atoms have the 1.2 fold isotropic displacement parameter of their bonding partners. Experimental details for each diffraction experiment are given in Table S1 (Supplementary Materials).

### 3.3. Computational Details

The geometry optimizations were performed at the BP86 level of theory, using Becke′s exchange functional [115] and Perdew′s correlation functional [116]. Ahlrich′s def2-SVP was chosen as basis set [117]. The Gaussian09 program package was used for all calculations [118]. Stationary points were characterized with frequency calculations at BP86/def2-SVP. The absolute energies and the Cartesian coordinates of the minimum structures of **6** and **6**′ are respectively given in Tables S2 and S3 (Supplementary Materials).

### 3.4. Synthetic Procedures

*1-tert-Butyl-3-tert-butylamino-4-methyl-1,2,4-triazol-4-ium tetrafluoroborate* (**6**H[BF_4_]). Dichloromethane (30 mL) was added to 1-*tert*-butyl-3-*tert*-butylamino-1,2,4-triazole (**5**) (6.50 g, 33.1 mmol) and trimethyloxonium tetrafluoroborate (5.00 g, 33.8 mmol). The suspension was stirred for 12 h. The resulting solution was filtered to remove traces of insoluble material. The filtrate was reduced to dryness *in vacuo*, leaving the product as a colorless microcrystalline solid. Yield 9.45 g (96%). ^1^H-NMR (400 MHz, acetone-*d*_6_, 25 °C): δ = 9.25 (s, 1H, NCHN), 6.04 (s, 1H, NH), 3.69 (s, 3H, NMe), 1.66, 1.45 (2 s, 2 × 9H CMe_3_) ppm. ^13^C-NMR (100 MHz, acetone-*d*_6_, 25 °C): δ = 153.7 (CN_3_), 137.9 (NCHN); 62.5, 53.5 (*C*Me_3_); 31.8 (NMe); 28.3, 28.2 (C*Me*_3_) ppm. HRMS/ESI(+): *m*/*z* = 211.1923 [M]^+^, 211.1917 calcd. for [C_11_H_23_N_4_]^+^. Anal. calcd. for C_11_H_23_N_4_BF_4_ (298.13): C 44.32, H 7.78, N 18.79; found: C 44.52, H 7.64, N 18.66.

*1-tert-Butyl-4-methyl-1,2,4-triazol-4-ium-3-tert-butylaminide* (**6**). THF (7 mL) and toluene (25 mL) were added to **6**H[BF_4_] (4.47 g, 15.0 mmol) and potassium *tert*-butoxide (1.68 g, 15.0 mmol). The suspension was stirred for 12 h. Insoluble material was separated from the liquid phase by centrifugation at 10 °C and subsequently extracted with toluene (2 × 10 mL). The liquid phase and extracts were combined and their volume subsequently reduced to ca. 30 mL *in vacuo*, then warmed up to ca. 100 °C and filtered through a short pad of Celite to remove insoluble material. The filtrate was reduced to dryness *in vacuo*. The solid was washed with *n*-hexane (10 mL) and dried *in vacuo*, leaving the product as a light yellow microcrystalline solid. Yield 2.87 g (91%). ^1^H-NMR (400 MHz, CD_2_Cl_2_, 25 °C): δ = 7.70 (s, 1H, NCHN), 3.31 (s, 3H, NMe), 1.52, 1.28 (2 s, 2 × 9H, CMe_3_) ppm. ^13^C-NMR (100 MHz, CD_2_Cl_2_, 25 °C): δ = 156.0 (CN_3_), 132.8 (NCHN); 59.4, 51.7 (*C*Me_3_); 30.0 (NMe); 29.5, 28.5 (C*Me*_3_) ppm. Anal. calcd. for C_11_H_22_N_4_ (210.32): C 62.82, H 10.54, N 26.64; found: C 60.82, H 10.56, N 25.36. Although the results for carbon and nitrogen are outside the range viewed as establishing analytical purity, these data are provided to illustrate the best values obtained to date. Very likely, the product contains small amounts of residual potassium tetrafluoroborate.

*Thione* **6** = S. Toluene (10 mL) was added to **6** (210 mg, 1.00 mmol) and elemental sulfur (33 mg, 0.13 mmol S_8_). The solution was stirred for 12 h and subsequently reduced to dryness *in vacuo*. The crude product was purified by sublimation at 130 °C and 10^−3^ mbar, which afforded a very pale yellow microcrystalline solid. Yield 225 mg (93%). ^1^H-NMR (400 MHz, CDCl_3_, 25 °C): δ = 3.39 (s, 1H, NH), 3.33 (s, 3H, NMe), 1.74, 1.37 (2 s, 2 × 9H, CMe_3_) ppm. ^13^C-NMR (100 MHz, CDCl_3_, 25 °C): δ = 161.3 (CS), 147.6 (CN_3_); 61.4, 52.5 (*C*Me_3_); 28.9 (NMe); 29.0, 28.2 (C*Me*_3_) ppm. HRMS/ESI(+): *m*/*z* = 243.1633 [M + H]^+^, 243.1643 calcd. for [C_11_H_23_N_4_S]^+^. Anal. calcd. for C_11_H_22_N_4_S (242.38): C 54.51, H 9.15, N 23.11, S 13.23; found: C 53.95, H 9.26, N 22.73, S 13.29.

*Selenone* **6** = Se. Toluene (10 mL) was added to **6** (105 mg, 0.50 mmol) and gray selenium powder (39 mg, 0.49 mmol). The mixture was stirred for 12 h and subsequently reduced to dryness *in vacuo*. The crude product was purified by sublimation at 130 °C and 10^−3^ mbar, which afforded a colorless microcrystalline solid. Yield 114 mg (80%). ^1^H-NMR (400 MHz, CDCl_3_, 25 °C): δ = 3.52 (s, 1H, NH), 3.48 (s, 3H, NMe), 1.79, 1.36 (2 s, 2 × 9H, CMe_3_) ppm. ^13^C-NMR (100 MHz, CDCl_3_, 25 °C): δ = 153.2 (CSe), 148.9 (CN_3_); 62.4, 52.5 (*C*Me_3_); 30.7 (NMe); 28.9, 28.6 (C*Me*_3_) ppm. ^77^Se NMR (95 MHz, CDCl_3_, 25 °C): δ = 89 ppm. ^77^Se NMR (95 MHz, acetone-*d*_6_, 25 °C): δ = 103 ppm. HRMS/ESI(+): *m*/*z* = 313.0901 [M + Na]^+^, 313.0907 calcd. for [C_11_H_22_N_4_NaSe]^+^. Anal. calcd. for C_11_H_22_N_4_Se (289.28): C 45.67, H 7.67, N 19.37; found: C 45.67, H 7.61, N 19.40.

*[RhCl(***6**′*)(COD)].* THF (5 mL) was added to **6** (112 mg, 0.53 mmol) and [{Rh(μ-Cl)(COD)}_2_] (125 mg, 0.25 mmol). The solution was stirred for 2 h. Volatile components were removed *in vacuo* and the solid residue subjected to purification by column chromatography (silica gel, dichloromethane to remove small amounts of unreacted starting materials, ethyl acetate for subsequent product elution), affording the product as yellow crystals. Yield 173 mg (75%). ^1^H-NMR (400 MHz, acetone-*d*_6_, 25 °C): δ = 5.11 (s, 1H, NH), 4.84 (m, 2H, CH), 4.07 (s, 3H, NMe), 3.31 (m, 2H, CH), 2.48, 2.36, 1.96 (3 m, 3 × 2H, CH_2_), 1.90 (s, 9H, CMe_3_), 1.84 (m, 2H, CH_2_), 1.38 (s, 9H, CMe_3_) ppm. ^13^C-NMR (100 MHz, acetone-*d*_6_, 25 °C): δ = 180.4 (d, ^1^*J*(^13^C,^103^Rh) 50.3 Hz, N_2_CRh), 152.7 (CN_3_), 96.5 (d, ^1^*J*(^13^C,^103^Rh) 7.8 Hz, CH), 94.6 (d, ^1^*J*(^13^C,^103^Rh) 7.4 Hz, CH), 70.0 (d, ^1^*J*(^13^C,^103^Rh) = 15.0 Hz, CH), 68.0 (d, ^1^*J*(^13^C,^103^Rh) = 14.2 Hz, CH); 60.5, 52.6 (*C*Me_3_); 34.5 (NMe); 33.9, 32.5 (CH_2_); 31.1 (C*Me*_3_); 29.9, 29.1 (CH_2_); 28.8 (C*Me*_3_) ppm. HRMS/ESI(+): *m*/*z* = 421.1831 [M − Cl]^+^, 421.1839 calcd. for [C_19_H_34_N_4_Rh]^+^. Anal. calcd. for C_19_H_34_N_4_ClRh (456.86): C 49.95, H 7.50, N 12.26; found: C 50.11, H 7.56, N 11.85.

*[RhCl(***6**′*)(CO)_2_].* The carbonyl complex was generated in situ on a small scale by subjecting a stirred solution of [RhCl(**6**′)(COD)] (30 mg, 0.07 mmol) in dichloromethane (3 mL) to an atmospheric pressure of carbon monoxide. The formation of the product was monitored by in situ FTIR spectroscopy. After completion of the reaction, which took only a few minutes, volatile components were removed *in vacuo*. The residue was dissolved in CD_2_Cl_2_ and the solution transferred to an NMR tube for further spectroscopic analysis. Yield quantitative (NMR). ^1^H-NMR (400 MHz, CD_2_Cl_2_, 25 °C): δ = 3.75 (s, 1H NH), 3.73 (s, 3H NMe), 1.74, 1.41 (2 s, 2 × 9H CMe_3_) ppm. ^13^C-NMR (100 MHz, CD_2_Cl_2_, 25 °C): δ = 186.4 (d, ^1^*J*(^13^C,^103^Rh) = 55.4 Hz, CO), 183.0 (d, ^1^*J*(^13^C,^103^Rh) = 74.7 Hz, CO), 171.4 (d, ^1^*J*(^13^C,^103^Rh) = 42.8 Hz, N_2_CRh), 151.7 (CN_3_); 60.8, 52.8 (*C*Me_3_); 34.3 (NMe); 31.0, 28.6 (C*Me*_3_) ppm. IR (CD_2_Cl_2_, 25 °C): ν_CO_ = 2081, 2003 cm^–1^.

*[CuCl(***6**′*)]*. THF (10 mL) was added to **6** (220 mg, 1.05 mmol) and copper(I) chloride (101 mg, 1.02 mmol). The suspension was stirred for 12 h and the resulting solution subsequently reduced to dryness *in vacuo*. The solid was washed with a mixture of diethyl ether and *n*-hexane (1:2, 15 mL), leaving the product as a colorless microcrystalline solid, which was dried *in vacuo*. Yield 254 mg (80%). ^1^H-NMR (400 MHz, CDCl_3_, 25 °C): δ = 3.94 (s, 1H NH), 3.59 (s, 3H NMe), 1.65, 1.38 (2 s, 2 × 9H CMe_3_) ppm. ^13^C-NMR (100 MHz, CDCl_3_, 25 °C): δ = 171.5 (N_2_CCu), 151.1 (CN_3_); 59.7, 52.7 (*C*Me_3_); 33.5 (NMe); 30.7, 28.6 (C*Me*_3_) ppm. HRMS/ESI(+): *m*/*z* = 331.0720 [M + Na]^+^, 331.0727 calcd. for [C_11_H_22_ClCuN_4_Na]^+^. Anal. calcd. for C_11_H_22_N_4_ClCu (309.32): C 42.71, H 7.17, N 18.11; found: C 42.70, H 7.04, N 17.88.

*[CuBr(***6**′*)]*. THF (10 mL) was added to **6** (226 mg, 1.07 mmol) and copper(I) bromide (144 mg, 1.00 mmol). The suspension was stirred for 12 h and the resulting solution subsequently reduced to dryness *in vacuo*. The solid was washed with a mixture of diethyl ether and *n*-hexane (1:2, 15 mL), leaving the product as a colorless microcrystalline solid, which was dried *in vacuo*. Yield 327 mg (92%). ^1^H-NMR (400 MHz, CDCl_3_, 25 °C): δ = 3.84 (s, 1H NH), 3.61 (s, 3H NMe), 1.66, 1.39 (2 s, 2 × 9H CMe_3_) ppm. ^13^C-NMR (100 MHz, CDCl_3_, 25 °C): δ = 172.8 (N_2_CCu), 151.0 (CN_3_); 59.8, 52.6 (*C*Me_3_), 33.5 (NMe); 30.7, 28.7 (C*Me*_3_) ppm. HRMS/ESI(+): *m*/*z* = 375.0205 [M + Na]^+^, 375.0222 calcd. for [C_11_H_22_BrCuN_4_Na]^+^. Anal. calcd. for C_11_H_22_N_4_BrCu (353.77): C 37.35, H 6.27, N 15.84; found: C 37.16, H 6.30, N 15.48.

*[CuI(***6**′*)]*. THF (10 mL) was added to **6** (221 mg, 1.05 mmol) and copper(I) iodide (192 mg, 1.01 mmol). The suspension was stirred for 12 h and the resulting solution subsequently reduced to dryness *in vacuo*. The solid was washed with a mixture of diethyl ether and *n*-hexane (1:2, 15 mL), leaving the product as a colorless microcrystalline solid, which was dried *in vacuo*. Yield 396 mg (98%). ^1^H-NMR (400 MHz, CDCl_3_, 25 °C): δ = 3.63 (s, 4H NH and NMe), 1.68, 1.40 (2 s, 2 × 9H CMe_3_) ppm. ^13^C-NMR (100 MHz, CDCl_3_, 25 °C): δ = 176.1 (N_2_CCu), 150.9 (CN_3_); 59.8, 52.6 (*C*Me_3_); 33.2 (NMe); 30.6, 28.7 (C*Me*_3_) ppm. Anal. calcd. for C_11_H_22_N_4_CuI (400.77): C 32.97, H 5.53, N 13.98; found: C 32.40, H 5.52, N 13.18.

*[AgCl(***6**′*)]*. THF (10 mL) was added to **6** (242 mg, 1.15 mmol) and AgCl (147 mg, 1.03 mmol). The suspension was stirred for 12 h and subsequently reduced to dryness *in vacuo*. The residue was dissolved in acetone (10 mL) and filtered through a short pad of Celite to remove trace amounts of insoluble material. The solvent was replaced by toluene (20 mL). The solution was filtered through a short pad of Celite to remove trace amounts of insoluble material. The volume of the filtrate was subsequently reduced to ca. 3 mL *in vacuo*. The product was precipitated by the addition of *n*-hexane (15 mL) and isolated by filtration as a colorless microcrystalline solid, which was dried *in vacuo*. Yield 323 mg (89%). ^1^H-NMR (400 MHz, acetone-*d*_6_, 25 °C): δ = 5.79 (s, 1H NH), 3.74 (s, 3H NMe), 1.70, 1.43 (2 s, 2 × 9H CMe_3_) ppm. ^13^C-NMR (100 MHz, acetone-*d*_6_, 25 °C): δ = 176.2 (N_2_CAg), 152.9 (CN_3_); 60.3, 52.9 (*C*Me_3_); 34.9 (NMe); 30.9, 28.6 (C*Me*_3_) ppm. HRMS/ESI(+): *m*/*z* = 375.0775 [M + Na]^+^, 375.0482 calcd. for [C_11_H_22_AgClN_4_Na]^+^. Anal. calcd. for C_11_H_22_N_4_AgCl (309.32): C 42.71, H 7.17, N 18.11; found: C 42.70, H 7.04, N 17.88. (353.64): C 37.36, H 6.27, N 15.84; found: C 37.33, H 6.22, N 15.77.

*[AgBr(***6**′*)]*. Toluene (5 mL) was added to **6** (215 mg, 1.02 mmol) and AgBr (191 mg, 1.02 mmol). The suspension was stirred for 12 h. The crude product was precipitated by the addition of diethyl ether (10 mL), filtered off and washed with diethyl ether (2 × 5 mL). The solid was dissolved in THF (7 mL) and filtered through a short pad of Celite to remove trace amounts of insoluble material. The filtrate was reduced to dryness *in vacuo*, leaving the product as a colorless microcrystalline solid, which was dried *in vacuo*. Yield 285 mg (71%). ^1^H-NMR (400 MHz, acetone-*d*_6_, 25 °C): δ = 5.48 (s, 1H NH), 3.71 (s, 3H NMe), 1.70, 1.43 (2 s, 2 × 9H CMe_3_) ppm. ^13^C-NMR (100 MHz, acetone-*d*_6_, 25 °C): δ = 177.5 (N_2_CAg), 152.8 (CN_3_); 60.3, 52.9 (*C*Me_3_); 34.7 (NMe); 30.9, 28.6 (C*Me*_3_) ppm. Anal. calcd. for C_11_H_22_N_4_AgBr (398.09): C 33.19, H 5.57, N 14.07; found: C 33.31, H 5.60, N 14.03.

*[AuCl(***6**′*)]*. THF (10 mL) was added to **6** (117 mg, 0.56 mmol) and [AuCl(THT)] (161 mg, 0.50 mmol). The suspension was stirred for 12 h. The resulting solution was filtered to remove a small amount of insoluble black material. The filtrate was reduced to dryness *in vacuo*. The solid washed with diethyl ether (2 × 5 mL), leaving the product as a colorless microcrystalline solid, which was dried *in vacuo*. Yield 160 mg (72%). The compound showed slow decomposition (indicated by a color change to light purple) even in the absence of light. Decomposition occurred significantly faster in solution, affording elemental gold and a yellow solution containing, *inter alia*, **6**H^+^ according to NMR spectroscopic analysis. ^1^H-NMR (500 MHz, CD_2_Cl_2_, 25 °C): δ = 4.02 (s, 1H NH), 3.61 (s, 3H NMe), 1.74, 1.40 (2 s, 2 × 9H CMe_3_) ppm. ^13^C-NMR (125 MHz, CD_2_Cl_2_, 25 °C): δ = 168.4 (N_2_CAu), 150.6 (CN_3_); 61.2, 53.0 (*C*Me_3_); 34.2 (NMe); 31.1, 28.6 (C*Me*_3_) ppm. HRMS/ESI(+): *m*/*z* = 465.1089 [M + Na]^+^, 465.1096 calcd. for [C_11_H_22_AuClN_4_Na]^+^. Due to the unstable nature of the product, satisfactory microanalytical data could not be obtained.

*[CuCl(***6**′*)_2_]*. Dichloromethane (10 mL) was added to **6** (225 mg, 1.07 mmol) and copper(I) chloride (49 mg, 0.49 mmol). The suspension was stirred for 12 h. The product was allowed to settle down and isolated as a colorless microcrystalline solid by decanting off the supernatant, followed by drying *in vacuo*. Yield 249 mg (97%). ^1^H-NMR (500 MHz, DMSO-*d*_6_, 70 °C): δ = 5.79 (s, 2H NH), 3.62 (s, 6H NMe), 1.64, 1.38 (2 s, 2 × 18H CMe_3_) ppm. ^13^C-NMR (125 MHz, DMSO-*d*_6_, 70 °C): δ = 174.1 (N_2_CCu), 151.3 (CN_3_); 58.6, 51.3 (*C*Me_3_); 32.8 (NMe); 29.8, 27.9 (C*Me*_3_) ppm. HRMS/ESI(+): *m*/*z* = 483.2970 [M − Cl]^+^, 483.2985 calcd. for [C_22_H_44_CuN_8_]^+^. Anal. calcd. for C_22_H_44_N_8_ClCu (519.64): C 50.85, H 8.53, N 21.56; found: C 50.69, H 8.54, N 21.16.

*[CuBr(***6**′*)_2_].* Dichloromethane (10 mL) was added to **6** (220 mg, 1.05 mmol) and copper(I) bromide (73 mg, 0.51 mmol). The suspension was stirred for 12 h. The product was allowed to settle down and isolated as a colorless microcrystalline solid by decanting off the supernatant, followed by drying *in vacuo*. Yield 279 mg (96%). ^1^H-NMR (500 MHz, DMSO-*d*_6_, 70 °C): δ = 5.81 (s, 2H NH), 3.64 (s, 6H NMe), 1.66, 1.38 (2 s, 2 × 18H CMe_3_) ppm. ^13^C-NMR (125 MHz, DMSO-*d*_6_, 70 °C): δ = 178.1 (N_2_CCu), 151.4 (CN_3_); 58.7, 51.3 (*C*Me_3_); 33.0 (NMe); 29.8, 27.9 (C*Me*_3_) ppm. HRMS/ESI(+): *m*/*z* = 483.2972 [M − Br]^+^, 483.2985 calcd. for [C_22_H_44_CuN_8_]^+^. Anal. calcd. for C_22_H_44_N_8_BrCu (564.09): C 46.84, H 7.86, N 19.86; found: C 46.97, H 7.83, N 19.64.

*[CuI(***6**′*)_2_].* Dichloromethane (10 mL) was added to **6** (219 mg, 1.04 mmol) and copper(I) iodide (95 mg, 0.49 mmol). The suspension was stirred for 12 h. The product was allowed to settle down and isolated as a colorless microcrystalline solid by decanting off the supernatant, followed by drying *in vacuo*. Yield 293 mg (97%). ^1^H-NMR (500 MHz, DMSO-*d*_6_, 70 °C): δ = 5.82 (s, 2H NH), 3.63 (s, 6H NMe), 1.67, 1.38 (2 s, 2 × 18H CMe_3_) ppm. ^13^C-NMR data determined *via* HSQC and HMBC (^1^H 500 MHz, decouple ^13^C 125 MHz; DMSO-*d*_6_, 70 °C): δ = 173.7 (N_2_CCu), 149.4 (CN_3_); 59.5, 52.1 (*C*Me_3_); 33.6 (NMe); 30.4, 28.5 (C*Me*_3_) ppm. HRMS/ESI(+): *m*/*z* = 483.2963 [M − I]^+^, 483.2985 calcd. for [C_22_H_44_CuN_8_]^+^. Anal. calcd. for C_22_H_44_N_8_CuI (611.09): C 43.24, H 7.26, N 18.34; found: C 43.13, H 7.29, N 18.32.

*[Ag(***6**′*)_2_]Cl*. THF (10 mL) was added to **6** (252 mg, 1.20 mmol) and AgCl (82 mg, 0.57 mmol). The suspension was stirred for 12 h and subsequently reduced to dryness *in vacuo*. The residue was dissolved in acetone (10 mL) and filtered through a short pad of Celite to remove trace amounts of insoluble material. The solvent was replaced by toluene (120 mL). The solution was filtered through a short pad of Celite to remove trace amounts of insoluble material. The volume of the filtrate was subsequently reduced to ca. 5 mL *in vacuo*. The product was precipitated by addition of *n*-hexane (15 mL) and isolated as a colorless microcrystalline solid, which was dried *in vacuo*. Yield 156 mg (48%). ^1^H-NMR (400 MHz, DMSO-*d*_6_, 25 °C): δ = 6.26 (s, 2H NH), 3.65 (s, 6H NMe), 1.64, 1.36 (2 s, 2 × 18H CMe_3_) ppm. ^13^C-NMR (100 MHz, DMSO-*d*_6_, 25°C): δ = 175.8 (N_2_CAg), 151.9 (CN_3_); 59.1, 51.6 (*C*Me_3_); 34.1 (NMe), 30.7, 28.0 (C*Me*_3_) ppm. HRMS/ESI(+): *m*/*z* = 527.2740 [M − Cl]^+^, 527.2740 calcd. for [C_22_H_44_AgN_8_]^+^. Anal. calcd. for C_22_H_44_N_8_AgCl (563.96): C 46.85, H 7.86, N 19.87; found: C 44.79, H 7.74, N 18.26. Although the results for carbon and nitrogen are outside the range viewed as establishing analytical purity, these data are provided to illustrate the best values obtained to date. The data are in accord with a mixture of the “2:1 complex” and the “1:1 complex” in a ratio of ca. 3:1. However, the NMR data do not show the presence of significant amounts of the “1:1 complex”.

*[Ag(***6**′*)_2_]Br*. THF (10 mL) was added to **6** (232 mg, 1.10 mmol) and AgBr (99 mg, 0.53 mmol). The suspension was stirred for 12 h and subsequently reduced to dryness *in vacuo*. The residue was dissolved in acetone (10 mL) and filtered through a short pad of Celite to remove trace amounts of insoluble material. The solvent was replaced by toluene (120 mL). The solution was filtered through a short pad of Celite to remove trace amounts of insoluble material. The volume of the filtrate was subsequently reduced to ca. 5 mL *in vacuo*. The product was precipitated by addition of *n*-hexane (15 mL) and isolated as a colorless microcrystalline solid, which was dried *in vacuo*. Yield 160 mg (50%). ^1^H-NMR (400 MHz, DMSO-*d*_6_, 25 °C): δ = 6.10 (s, 2H NH), 3.64 (s, 6H NMe), 1.64, 1.37 (2 s, 2 × 18H CMe_3_) ppm. ^13^C-NMR (100 MHz, DMSO-*d*_6_, 25°C): δ = 176.5 (N_2_CAg), 151.8 (CN_3_); 59.1, 51.6 (*C*Me_3_); 34.0 (NMe); 30.1, 28.1 (C*Me*_3_) ppm. Anal. calcd. for C_22_H_44_N_8_AgBr (608.41): C 43.43, H 7.29, N 18.42; found: C 43.43, H 7.22, N 17.69.

*[Ag(***6**′*)_2_](OTf)*. Dichloromethane (10 mL) was added to **6** (186 mg, 0.88 mmol) and silver triflate (108 mg, 0.42 mmol). The solution was stirred for 12 h and subsequently reduced to dryness *in vacuo*. The residue was washed with diethyl ether (5 mL) and dried *in vacuo*, leaving the product as a colorless microcrystalline solid. Yield 264 mg (93%). ^1^H-NMR (400 MHz, CDCl_3_, 25 °C): δ = 3.64 (s, 6H NMe), 1.64, 1.38 (2 s, 2 × 18H CMe_3_) ppm. The NH signal could not be detected. ^13^C-NMR (100 MHz, CDCl_3_, 25 °C): δ = 173.3 (d/d, ^1^*J*(^13^C,^109/107^Ag) = 213/190 Hz, CAg), 151.0 (CN_3_), 119.7 (q, ^1^*J*(^13^C,^19^F) = 320 Hz, CF_3_); 58.6, 51.3 (*C*Me_3_); 33.2 (NMe); 29.7, 27.2 (C*Me*_3_) ppm. HRMS/ESI(+): *m*/*z* = 527.2708 [Ag(**6**′)_2_]^+^, 527.2740 calcd. for [C_22_H_44_AgN_8_]^+^. Anal. calcd. for C_23_H_44_N_8_AgF_3_O_3_S (677.58): C 40.77, H 6.55, N 16.54, S 4.73; found: C 40.90, H 6.60, N 16.38, S 4.67.

*[Au(***6**′*)_2_]Cl*. THF (5 mL) was added to **6** (218 mg, 1.04 mmol) and [AuCl(THT)] (157 mg, 0.49 mmol). The solution was stirred for 15 min and subsequently reduced to dryness *in vacuo*. The residue was washed with diethyl ether (5 mL) and *n*-hexane (2 × 5 mL). The crude product was dissolved in toluene (5 mL) and filtered through a short pad of Celite to remove trace amounts of insoluble material. The product was precipitated from the filtrate by addition of *n*-hexane (15 mL) and isolated as a colorless microcrystalline solid, which was dried *in vacuo*. Yield 237 mg (74%). ^1^H-NMR (500 MHz, CD_2_Cl_2_, 25 °C): δ = 5.43 (s, 2H NH), 3.76 (s, 6H NMe), 1.74, 1.42 (2 s, 2 × 18H CMe_3_) ppm. ^13^C-NMR (100 MHz, CD_2_Cl_2_, 25 °C): δ = 179.9 (N_2_CAu), 152.0 (CN_3_); 61.0, 52.8 (*C*Me_3_); 34.6 (NMe); 31.2, 28.5 (C*Me*_3_) ppm. HRMS/ESI(+): *m*/*z* = 617.3325 [M − Cl]^+^, 617.3355 calcd. for [C_22_H_44_AuN_8_]^+^. Anal. calcd. for C_22_H_44_N_8_AuCl (653.06): C 40.46, H 6.79, N 17.16; found: C 40.54, H 6.76, N 17.05.

## 4. Conclusions

The conjugated mesomeric betaine 1-*tert*-butyl-4-methyl-1,2,4-triazol-4-ium-3-*tert*-butylaminide (**6**) is an “instant carbene” analogous to Nitron (**2**), reacting smoothly and swiftly with typical carbene-trapping reagents under mild conditions. In particular, precious metal complexes of its carbenic tautomer 1-*tert*-butyl-3-*tert*-butylamino-4-methyl-1,2,4-triazol-5-ylidene (**6**′) are easily obtained. **6**′ is less electrophilic and a better σ-donor than the carbenic tautomer of Nitron (**2**′). Consequently, the net electron donor capacity of **6**′ is superior to that of **2**′. This is due to the alkyl substituents of **6**′, which are more electron-donating than the phenyl substituents of **2**′. A comparison of the complexes of **2**′ and **6**′ reveals two pronounced structural differences. First, [CuX(**6**′)_2_] (X = Cl, Br) exhibit more acute C–Cu–C bond angles than [CuX(**2**′)_2_]. Second, [CuCl(**6**′)] forms dimeric aggregates through Cu···Cu contacts of ca. 2.87 Å, compatible with cuprophilic interactions. These differences may be explained by the complementary steric requirements of the *tert*-butyl and the methyl substituent of **6**′, which allow a comparatively closer approach of two such ligands, both intra- and intermolecularly.

## Supplementary Materials

Supplementary materials are available online. Tables S1–S4: details concerning crystallographic and computational work, Figures S1–S40: plots of NMR spectra. CCDC 1558100–1558111 contain the supplementary crystallographic data for this paper. These data can be obtained free of charge via http://www.ccdc.cam.ac.uk/conts/retrieving.html (or from the CCDC, 12 Union Road, Cambridge CB2 1EZ, UK; Fax: +44 1223 336033; E-mail: deposit@ccdc.cam.ac.uk).
