# Supplementary material for: Coinage Metal Complexes of the Carbenic Tautomer of A Conjugated Mesomeric Betaine Akin to Nitron
Source: Molecules. 2017 Jul 7;22(7):1133. doi: 10.3390/molecules22071133 (PMC6152182; doi:10.3390/molecules22071133)
Supplement: Supplementary file 1 [file molecules-22-01133-s001.pdf]

Supplementary Materials: Coinage Metal Complexes of the Carbenic Tautomer of a Conjugated Mesomeric Betaine Akin to Nitron

Charlotte Thie, Clemens Bruhn, Michael Leibold and Ulrich Siemeling

X-ray crystallography:

Table S1. Data Collection and Structure Refinement Details. S2

DFT calculations:

Table S2. Absolute energy and Cartesian coordinates (Å) for the minimum structure of **6**. S3
Table S3. Absolute energy and Cartesian coordinates (Å) for the minimum structure of **6'**. S4
Table S4. Experimentally determined and DFT computed bond lengths (Å) and angles (°) of **6**. S4

Plots of NMR spectra:

Figure S1. ^{1}H NMR spectrum of **6**H[BF_4] (acetone-d_6, 400 MHz). S5
Figure S2. ^{13}C NMR spectrum of **6**H[BF_4] (acetone-d_6, 100 MHz). S5
Figure S3. ^{1}H NMR spectrum of **6** (CD_2Cl_2, 400 MHz). S6
Figure S4. ^{13}C NMR spectrum of **6** (CD_2Cl_2, 100 MHz). S6
Figure S5. ^{1}H NMR spectrum of **6'**=S ($CDCl_3$, 400 MHz). S7
Figure S6. ^{13}C NMR spectrum of **6'**=S ($CDCl_3$, 100 MHz). S7
Figure S7. ^{1}H NMR spectrum of **6'**=Se ($CDCl_3$, 400 MHz). S8
Figure S8. ^{13}C NMR spectrum of **6'**=Se ($CDCl_3$, 100 MHz). S8
Figure S9. ^{77}Se NMR spectrum of **6'**=Se ($CDCl_3$, 95 MHz). S9
Figure S10. ^{1}H NMR spectrum [RhCl(**6'**)(COD)] (acetone-d_6, 400 MHz). S9
Figure S11. ^{13}C NMR spectrum of [RhCl(**6'**)(COD)] (acetone-d_6, 100 MHz). S10
Figure S12. ^{1}H NMR spectrum of [RhCl(**6'**)$(CO)_2$] (CD_2Cl_2, 400 MHz). S10
Figure S13. ^{13}C NMR spectrum of [RhCl(**6'**)$(CO)_2$] (CD_2Cl_2, 100 MHz). S11
Figure S14. ^{1}H NMR spectrum of [CuCl(**6'**)] ($CDCl_3$, 400 MHz). S11
Figure S15. ^{13}C NMR spectrum of [CuCl(**6'**)] ($CDCl_3$, 100 MHz). S12
Figure S16. ^{1}H NMR spectrum of [CuBr(**6'**)] ($CDCl_3$, 400 MHz). S12
Figure S17. ^{13}C NMR spectrum of [CuBr(**6'**)] ($CDCl_3$, 100 MHz). S13
Figure S18. ^{1}H NMR spectrum of [CuI(**6'**)] ($CDCl_3$, 400 MHz). S13
Figure S19. ^{13}C NMR spectrum of [CuI(**6'**)] ($CDCl_3$, 100 MHz). S14
Figure S20. ^{1}H NMR spectrum of [AgCl(**6'**)] (acetone-d_6, 400 MHz). S14
Figure S21. ^{13}C NMR spectrum of [AgCl(**6'**)] (acetone-d_6, 100 MHz). S15
Figure S22. ^{1}H NMR spectrum of [AgBr(**6'**)] (acetone-d_6, 400 MHz). S15
Figure S23. ^{13}C NMR spectrum of [AgBr(**6'**)] (acetone-d_6, 100 MHz). S16
Figure S24. ^{1}H NMR spectrum of [AuCl(**6'**)] (CD_2Cl_2, 500 MHz). S16
Figure S25. ^{13}C NMR spectrum of [AuCl(**6'**)] (CD_2Cl_2, 125 MHz). S17
Figure S26. ^{1}H NMR spectrum of [CuCl$(\mathbf{6'})_2$] (DMSO-d_6, 500 MHz, 70 °C). S17
Figure S27. ^{13}C NMR spectrum of [CuCl$(\mathbf{6'})_2$] (DMSO-d_6, 125 MHz, 70 °C). S18
Figure S28. ^{1}H NMR spectrum of [CuBr$(\mathbf{6'})_2$] (DMSO-d_6, 500 MHz, 70 °C). S18
Figure S29. ^{13}C NMR spectrum of [CuBr$(\mathbf{6'})_2$] (DMSO-d_6, 125 MHz, 70 °C). S19
Figure S30. ^{1}H NMR spectrum of [CuI$(\mathbf{6'})_2$] (DMSO-d_6, 500 MHz, 70 °C). S19
Figure S31. HSQC NMR spectrum of [CuI$(\mathbf{6'})_2$] (DMSO-d_6, 500 MHz, 70 °C). S20
Figure S32. HMBC NMR spectrum of [CuI$(\mathbf{6'})_2$] (DMSO-d_6, 500 MHz, 70 °C). S21
Figure S33. ^{1}H NMR spectrum of [Ag$(\mathbf{6'})_2$]Cl (DMSO-d_6, 400 MHz). S22
Figure S34. ^{13}C NMR spectrum of [Ag$(\mathbf{6'})_2$]Cl (DMSO-d_6, 100 MHz). S22
Figure S35. ^{1}H NMR spectrum of [Ag$(\mathbf{6'})_2$]Br (DMSO-d_6, 400 MHz). S23
Figure S36. ^{13}C NMR spectrum of [Ag$(\mathbf{6'})_2$]Br (DMSO-d_6, 100 MHz). S23
Figure S37. ^{1}H NMR spectrum of [Ag$(\mathbf{6'})_2$](OTf) ($CDCl_3$, 400 MHz). S24
Figure S38. ^{13}C NMR spectrum of [Ag$(\mathbf{6'})_2$](OTf) ($CDCl_3$, 100 MHz). S24
Figure S39. ^{1}H NMR spectrum of [Au$(\mathbf{6'})_2$]Cl (CD_2Cl_2, 500 MHz). S25
Figure S40. ^{13}C NMR spectrum of [Au$(\mathbf{6'})_2$]Cl (CD_2Cl_2, 125 MHz). S25

Table S1. Data collection and structure refinement details.

	**6**H[BF$_4$]	**6**	**6‘=S**	[RhCl(**6‘**)(COD)]]	[RhI(**6‘**)(COD)]]	[CuCl(**6’**)]·CH$_2$Cl$_2$	[AuCl(**6’**)]	[CuCl(**6’**)$_2$]	[CuBr(**6’**)$_2$]	[AgBr(**6’**)$_2$]·C$_7$H$_8$	[Ag(**6’**)$_2$]Br	[Ag(**6’**)$_2$](OTf)
Chem. formula	$C_{11}H_{23}BF_4N_4$	$C_{11}H_{22}N_4$	$C_{11}H_{22}N_4S$	$C_{19}H_{34}ClN_4Rh$	$C_{19}H_{34}IN_4Rh$	$C_{23}H_{46}Cl_4Cu_2N_8$	$C_{11}H_{22}AuClN_4$	$C_{22}H_{44}ClCuN_8$	$C_{22}H_{44}BrCuN_8$	$C_{29}H_{52}AgBrN_8$	$C_{22}H_{44}AgBrN_8$	$C_{23}H_{44}AgF_3N_8O_3S$
Formula mass	298.14	210.32	242.38	456.86	548.31	703.56	442.74	519.64	564.10	700.56	608.43	677.59
Crystal system	orthorhombic	monoclinic	monoclinic	monoclinic	monoclinic	triclinic	monoclinic	orthorhombic	orthorhombic	monoclinic	orthorhombic	triclinic
Space group	*P b c a*	*I a*	*C* 2/*c*	*P* 2$_1$/*c*	*P* 2$_1$/*c*	*P* −1	*C* 2/*c*	*P b c n*	*P b c n*	*P* 2$_1$/*n*	*C* 2 2 2$_1$	*P* 1
a/Å	10.3118(3)	9.5203(5)	22.431(2)	13.2122(4)	13.5955(4)	10.3279(6)	15.3460(9)	10.9369(6)	10.8319(7)	14.7525(7)	9.3230(8)	8.2561(9)
b/Å	26.4187(10)	11.1150(5)	11.3451(6)	26.5736(9)	27.3237(10)	14.0466(8)	20.7976(9)	10.0225(6)	9.9667(6)	13.3286(4)	14.0077(9)	8.1726(9)
c/Å	11.3460(3)	11.7903(7)	11.3470(9)	12.1591(4)	12.1111(4)	13.9264(7)	11.7713(6)	24.9910(16)	24.815(2)	17.2248(7)	22.283(2)	12.9281(14)
α/°	90	90	90	90	90	65.343(4)	90	90	90	90	90	99.363(8)
β/°	90	90.563(4)	101.364(6)	103.994(3)	104.372(2)	84.903(5)	109.632(4)	90	90	95.498(4)	90	100.770(8)
γ/°	90	90	90	90	90	73.737(5)	90	90	90	90	90	112.147(8)
V/Å^3	3090.9(2)	1247.57(11)	2831.0(4)	4142.3(2)	4358.2(3)	1761.7(2)	3538.5(3)	2739.4(3)	2679.0(3)	3371.3(2)	2910.0(4)	767.22(15)
T/K	100(2)	100(2)	100(2)	100(2)	100(2)	100(2)	100(2)	100(2)	100(2)	100(2)	100(2)	100(2)
Crystal size/mm	0.55×0.32×0.08	0.15×0.15×0.05	0.32×0.09×0.02	0.19×0.17×0.07	0.27×0.17×0.08	0.23×0.04×0.02	0.03×0.03×0.02	0.41×0.24×0.04	0.18×0.14×0.02	0.18×0.17×0.12	0.25×0.17×0.05	0.30×0.05×0.05
Z	8	4	8	8	8	2	8	4	4	4	4	1
μ/mm^{-1}	0.112	0.544	0.212	0.963	2.210	1.535	8.455	0.919	2.332	1.813	2.089	0.781
No. of refls measured	23156	7774	6619	21672	27319	12584	7888	9413	6739	15506	12905	16235
Indep. refls	4668	1983	2631	7869	10239	6505	3241	2684	2484	6260	3501	5475
[R_{int}]	[0.0202]	[0.0109]	[0.0596]	[0.0364]	[0.0357]	[0.0434]	[0.0286]	[0.0409]	[0.0383]	[0.0608]	[0.0357]	[0.0316]
Final R_1 (wR_2) [$I > 2\sigma(I)$]	0.0382 (0.0885)	0.0250 (0.0637)	0.0689 (0.1853)	0.0580 (0.1593)	0.0457 (0.1240)	0.0441 (0.1020)	0.0347 (0.0862)	0.0884 (0.2423)	0.0584 (0.1519)	0.0809 (0.2016)	0.0298 (0.0638)	0.0709 (0.1881)
Final R_1 (wR_2) [all data]	0.0554 (0.0965)	0.0251 (0.0639)	0.0854 (0.2015)	0.0636 (0.1656)	0.0514 (0.1308)	0.0607 (0.1092)	0.0430 (0.0922)	0.1056 (0.2636)	0.0773 (0.1733)	0.1003 (0.2277)	0.0357 (0.0657)	0.0724 (0.1975)
Abs. correction	integration	integration	integration	integration	integration	integration	integration	integration	integration	integration	integration	integration
T_{min}/T_{max}	0.9595/0.9912	0.9071/0.9765	0.9685/0.9949	0.8472/0.9420	0.6364/0.8411	0.8217/0.9699	0.6176/0.8240	0.7717/0.9602	0.7309/0.9447	0.7481/0.8406	0.6168/0.8909	0.8660/0.9702
Gof on F^2	1.017	1.056	1.060	1.088	1.046	1.036	1.027	1.101	1.101	1.100	1.036	1.068
CCDC number	1558100	1558101	1558102	1558103	1558104	1558105	1558106	1558107	1558108	1558109	1558110	1558111

Table S2. Absolute energy and Cartesian coordinates (Å) for the minimum structure of **6**.

E(BP86/def2-SVP) = -650.918215 a.u.

C	-4.660469	0.465659	7.937394
N	-3.625286	0.401085	8.980282
C	-3.538653	1.638722	9.522881
N	-4.414668	2.424915	8.880850
N	-5.111141	1.763501	7.913318
C	-2.801037	-0.720100	9.350632
C	-4.697040	3.807805	9.166298
N	-4.965336	-0.594035	7.257957
C	-5.903369	-0.654745	6.167082
H	-2.946719	1.918577	10.398441
C	-5.972344	-2.012909	5.663959
C	-7.213822	-0.244810	6.633094
C	-5.468490	0.232351	5.105744
H	-7.923209	-0.290722	5.808121
H	-7.540431	-0.911050	7.430196
H	-7.162019	0.775218	7.010958
H	-6.177877	0.186439	4.280770
H	-5.416687	1.252379	5.483607
H	-4.484295	-0.075524	4.755753
H	-6.681732	-2.058821	4.838985
H	-4.988149	-2.320785	5.313967
H	-6.298954	-2.679149	6.461060
H	-3.083098	-1.589326	8.758341
H	-1.755360	-0.478654	9.165742
H	-2.941315	-0.940215	10.407888
C	-5.705343	4.295312	8.245341
C	-5.184686	3.930806	10.526287
C	-3.485424	4.589792	9.014695
H	-5.918887	5.341123	8.461211
H	-5.339104	4.202934	7.223942
H	-6.615309	3.708013	8.359200
H	-5.398230	4.976617	10.742157
H	-6.094652	3.343507	10.640146
H	-4.427416	3.564672	11.217958
H	-3.698968	5.635602	9.230565
H	-2.728154	4.223657	9.706366
H	-3.119185	4.497414	7.993296

Table S3. Absolute energy and Cartesian coordinates (Å) for the minimum structure of **6'**.

```
E(BP86/def2-SVP) = -650.911770 a.u.
 C    -4.592134     0.526051     8.006838
 N    -3.595388     0.465005     9.004936
 C    -3.453268     1.702220     9.609040
 N    -4.397342     2.419489     8.947966
 N    -5.111994     1.733750     7.958336
 C    -2.797617    -0.684890     9.343907
 C    -4.711355     3.804160     9.188135
 N    -4.890736    -0.553083     7.214733
 C    -5.862571    -0.672968     6.158909
 H    -4.296043    -1.371211     7.368457
 C    -5.814242    -2.013115     5.607393
 C    -7.187229    -0.416284     6.689846
 C    -5.564825     0.289778     5.116331
 H    -6.299775     0.199115     4.317864
 H    -5.601121     1.296275     5.530539
 H    -4.569961     0.097000     4.717579
 H    -7.922179    -0.506946     5.891379
 H    -7.410846    -1.139339     7.472857
 H    -7.223525     0.590213     7.104054
 H    -6.549192    -2.103778     4.808925
 H    -4.819379    -2.205893     5.208640
 H    -6.037860    -2.736170     6.390404
 H    -3.101823    -1.531452     8.730138
 H    -1.746821    -0.463952     9.162423
 H    -2.940891    -0.928876    10.395508
 C    -5.770910     4.222156     8.290848
 C    -5.147266     3.969813    10.561103
 C    -3.532084     4.614799     8.954290
 H    -6.008382     5.269313     8.472476
 H    -5.443526     4.097745     7.259702
 H    -6.656583     3.613338     8.466473
 H    -3.769556     5.661957     9.135918
 H    -2.736322     4.300870     9.628184
 H    -3.204700     4.490389     7.923144
 H    -5.384738     5.016970    10.742731
 H    -6.032939     3.360995    10.736728
 H    -4.351503     3.655884    11.234996
```

Table S4. Experimentally determined and DFT computed bond lengths (Å) and angles (°) of **6**.

	**XRD**	**DFT**
C1–N2	1.315(2)	1.337
C1–N3	1.339(2)	1.352
N2–N1	1.383(2)	1.365
C2–N4	1.308(2)	1.292
C2–N3	1.420(2)	1.453
C2–N1	1.364(2)	1.385
N1–C1–N3	107.46(13)	107.30

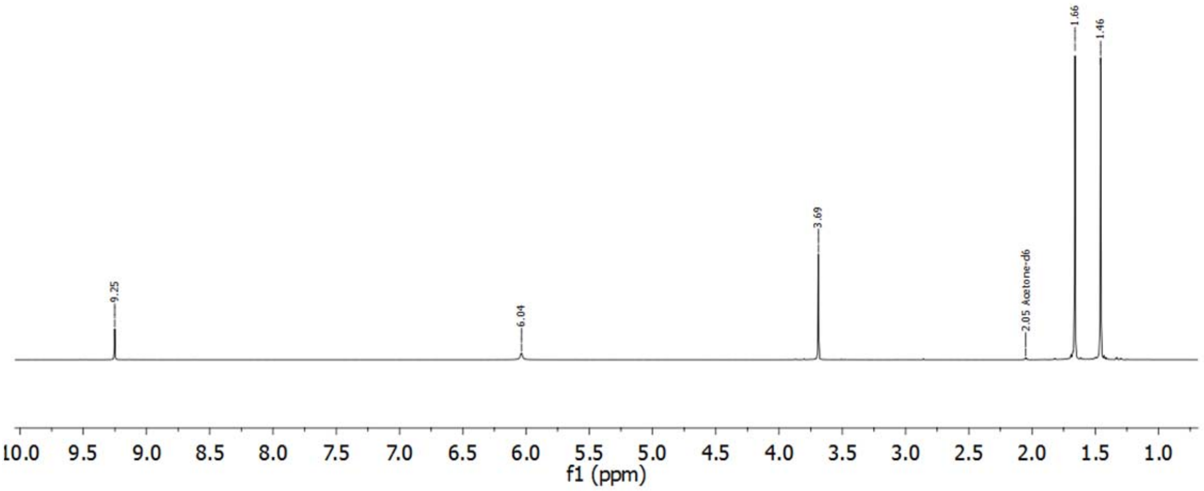

Figure S1. ^{1}H NMR spectrum of **6**H[BF_4] (acetone-d_6, 400 MHz).

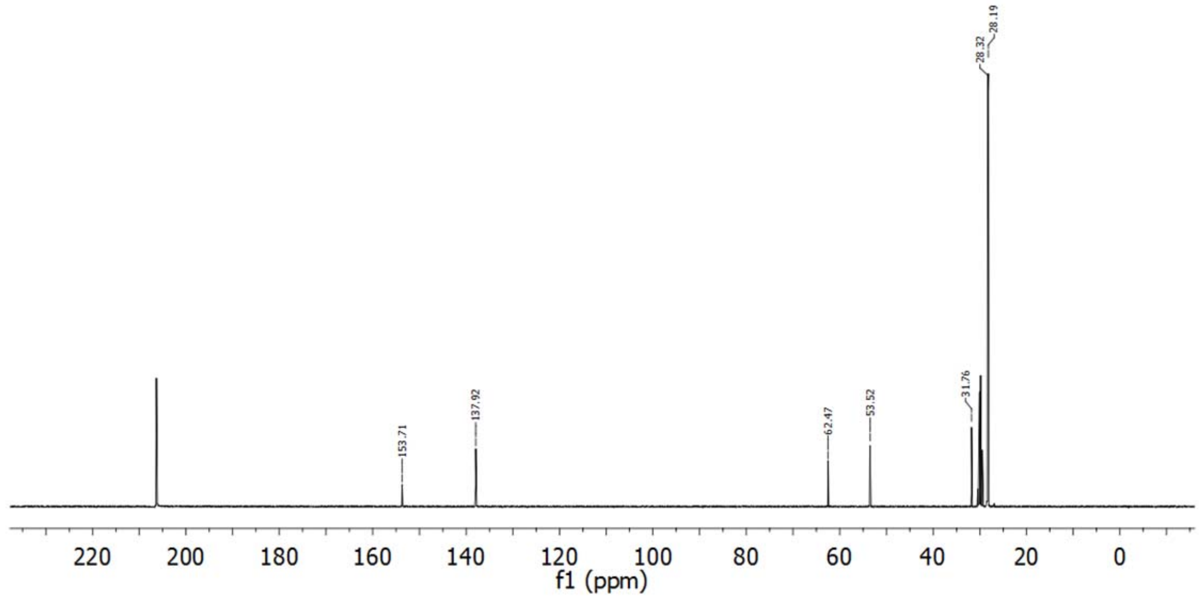

Figure S2. ^{13}C NMR spectrum of **6**H[BF_4] (acetone-d_6, 100 MHz).

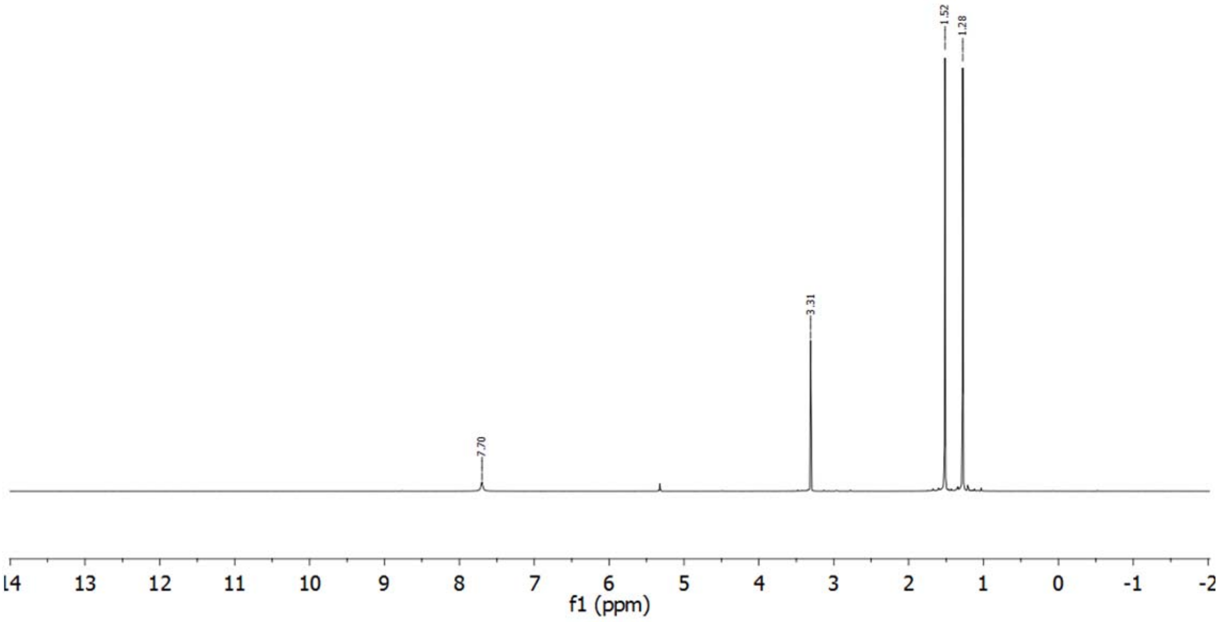

Figure S3. ^{1}H NMR spectrum of **6** (CD_2Cl_2, 400 MHz).

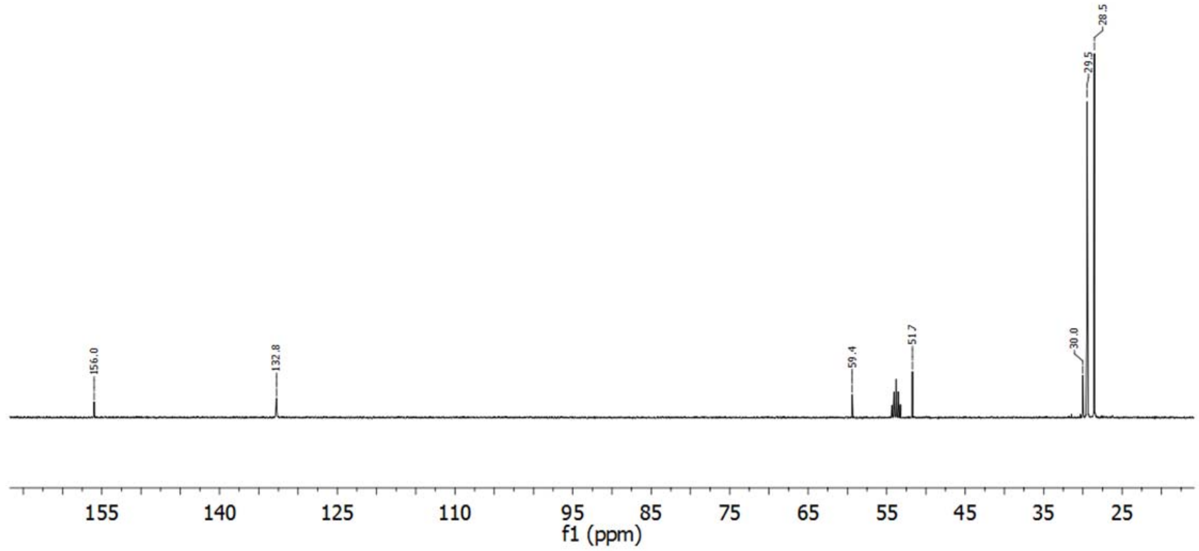

Figure S4. ^{13}C NMR spectrum of **6** (CD_2Cl_2, 100 MHz).

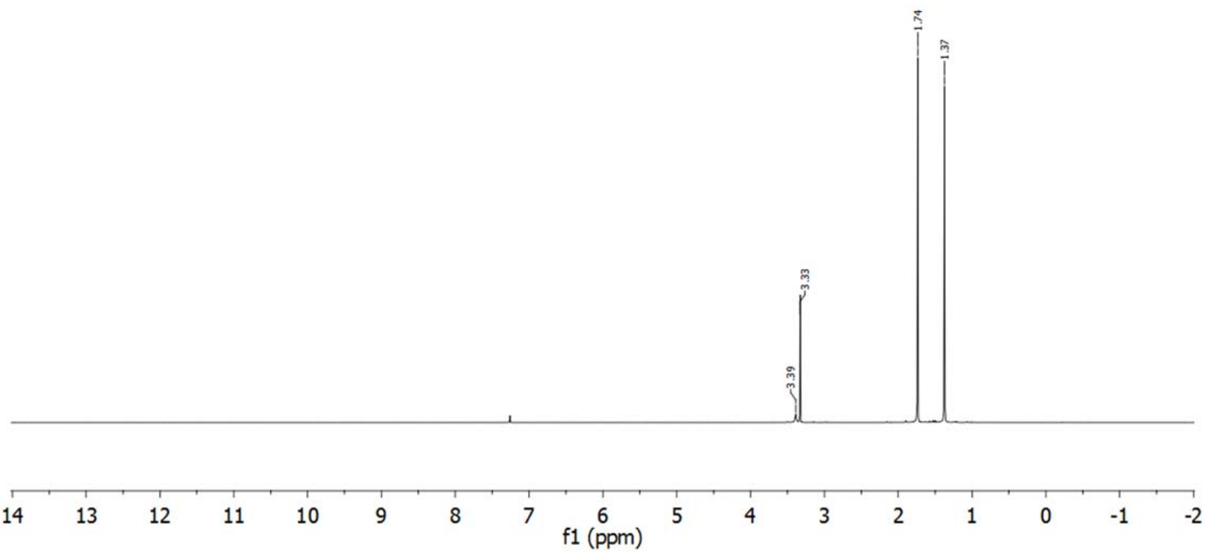

Figure S5. ^{1}H NMR spectrum of **6’**=S ($CDCl_3$, 400 MHz).

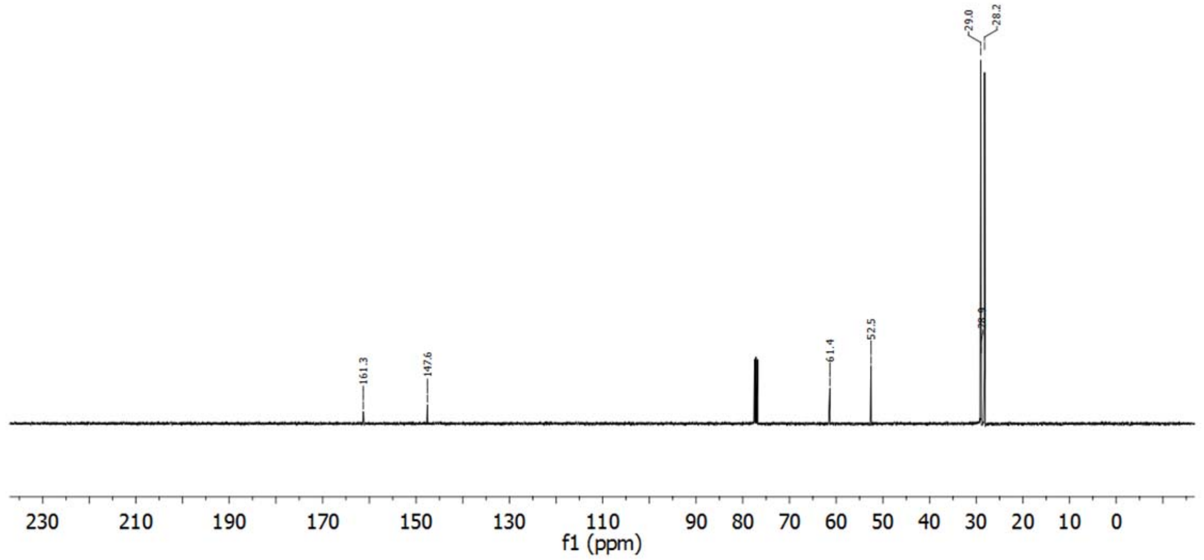

Figure S6. ^{13}C NMR spectrum of **6’**=S ($CDCl_3$, 100 MHz).

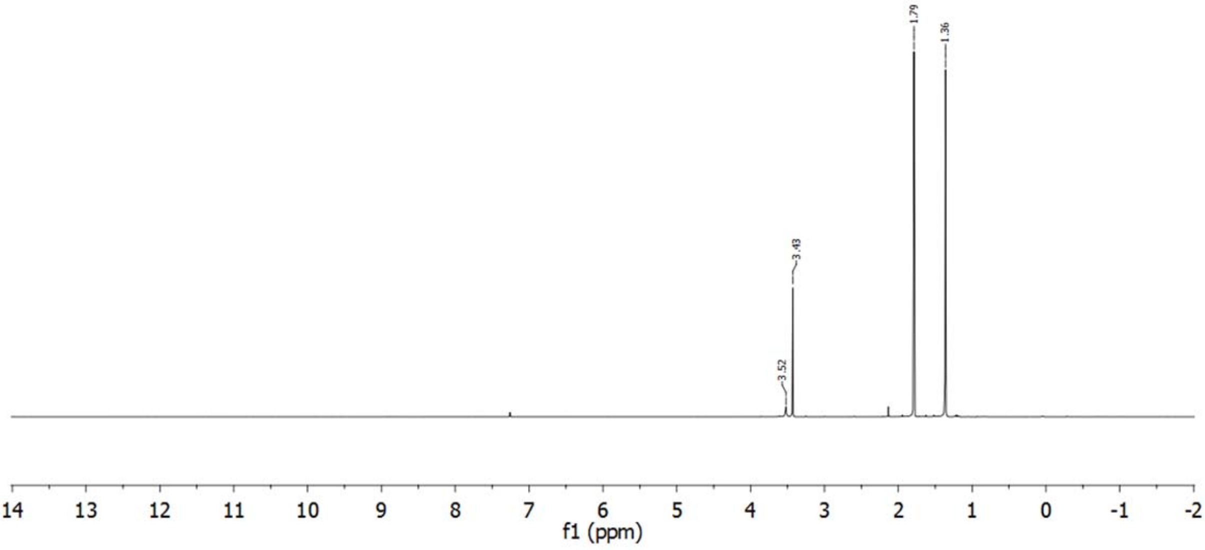

Figure S7. ^{1}H NMR spectrum of **6’**=Se ($CDCl_3$, 400 MHz). The signal at 2.1 ppm is due to acetone.

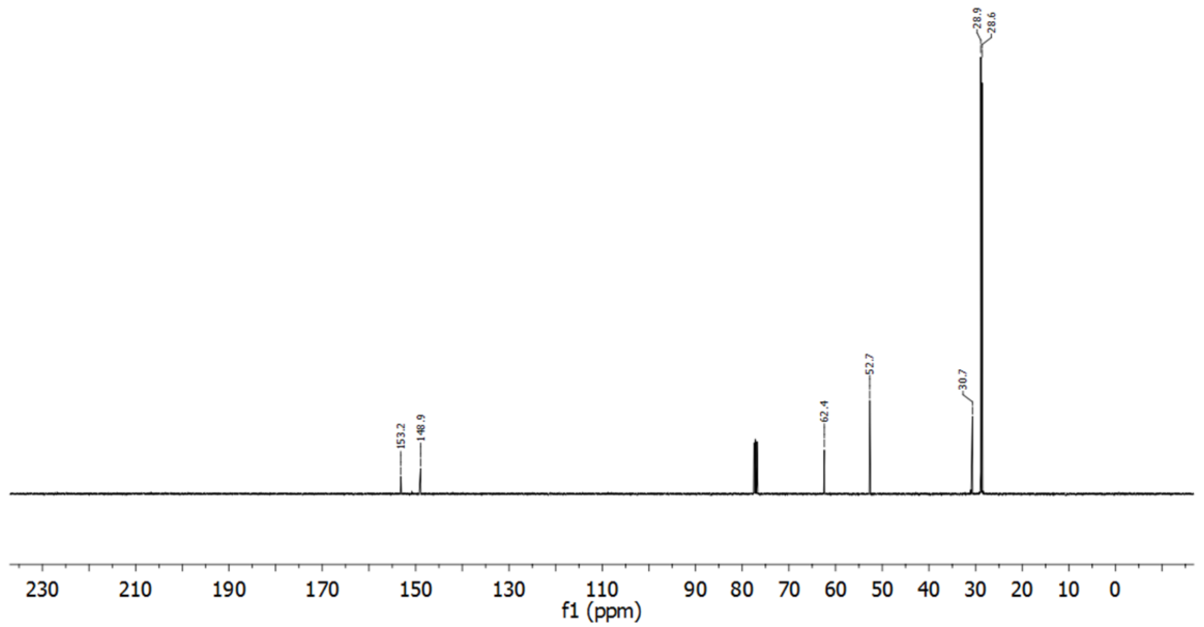

Figure S8. ^{13}C NMR spectrum of **6’**=Se ($CDCl_3$, 100 MHz).

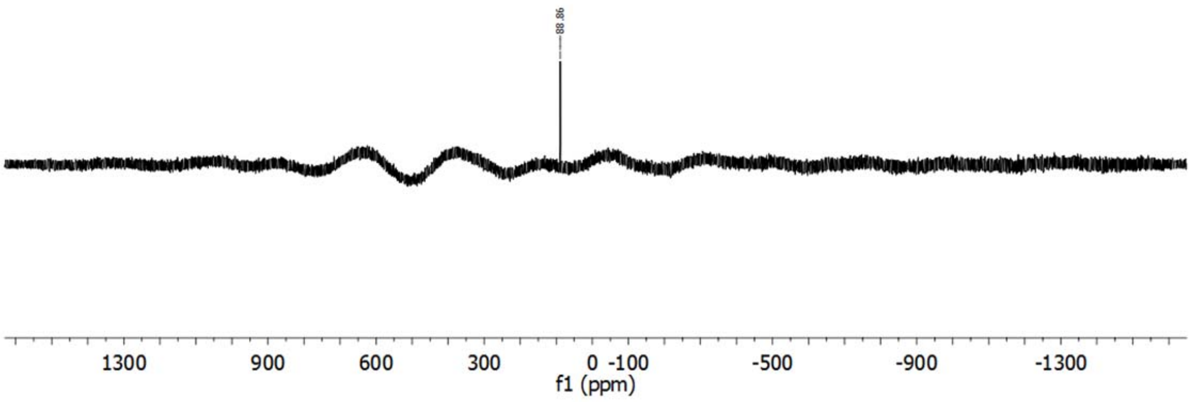

Figure S9. ^{77}Se NMR spectrum of **6’**=Se ($CDCl_3$, 95 MHz).

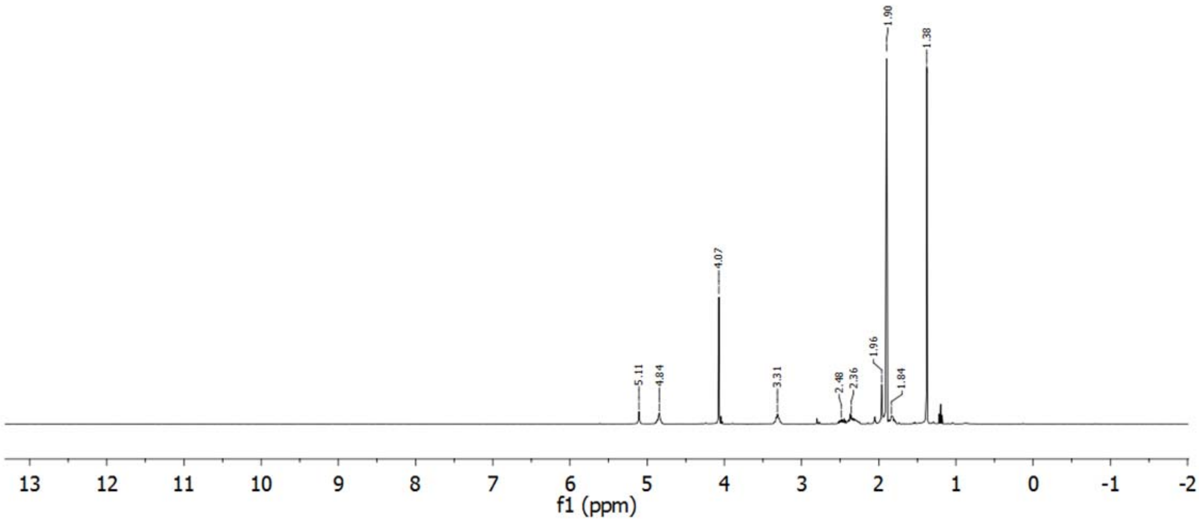

Figure S10. ^{1}H NMR spectrum [RhCl(**6’)**(COD)] (acetone-d_6, 400 MHz). The minor signals at 1.1 ppm and 2.7 ppm are due to an unknown impurity.

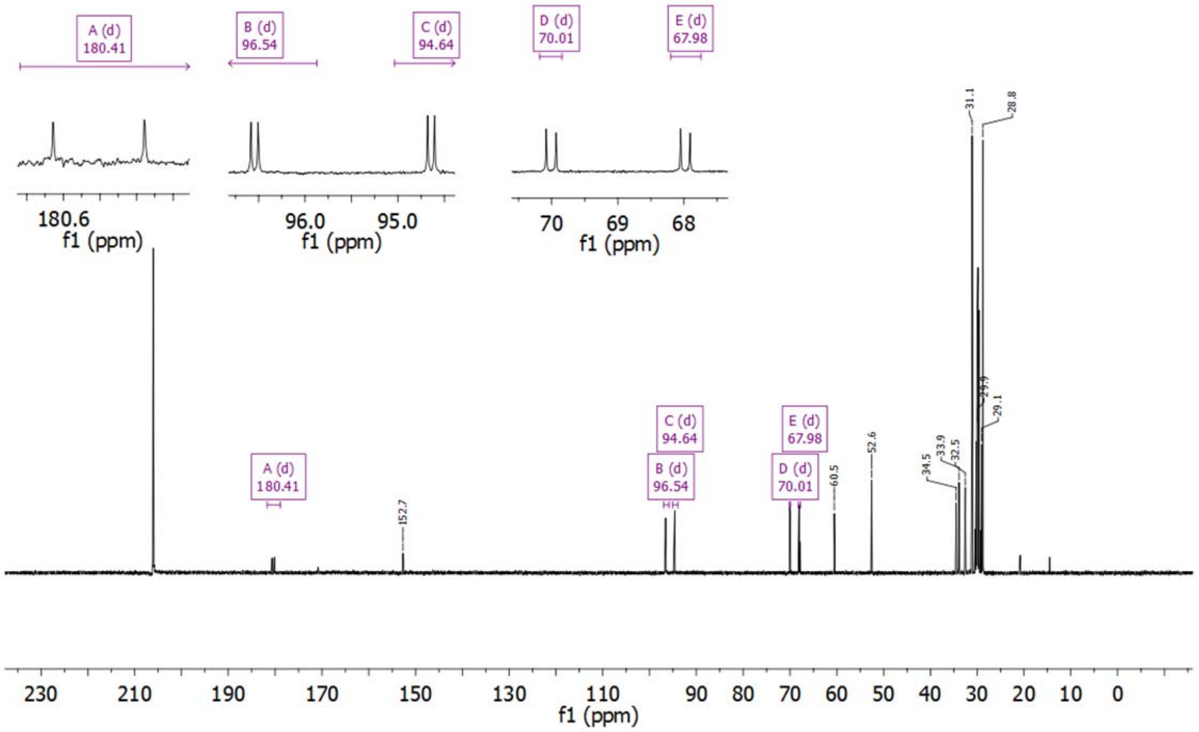

Figure S11. ^{13}C NMR spectrum of [RhCl(**6'**)(COD)] (acetone-d_6, 100 MHz). The minor signals at 21 and 15 ppm are due to an unknown impurity.

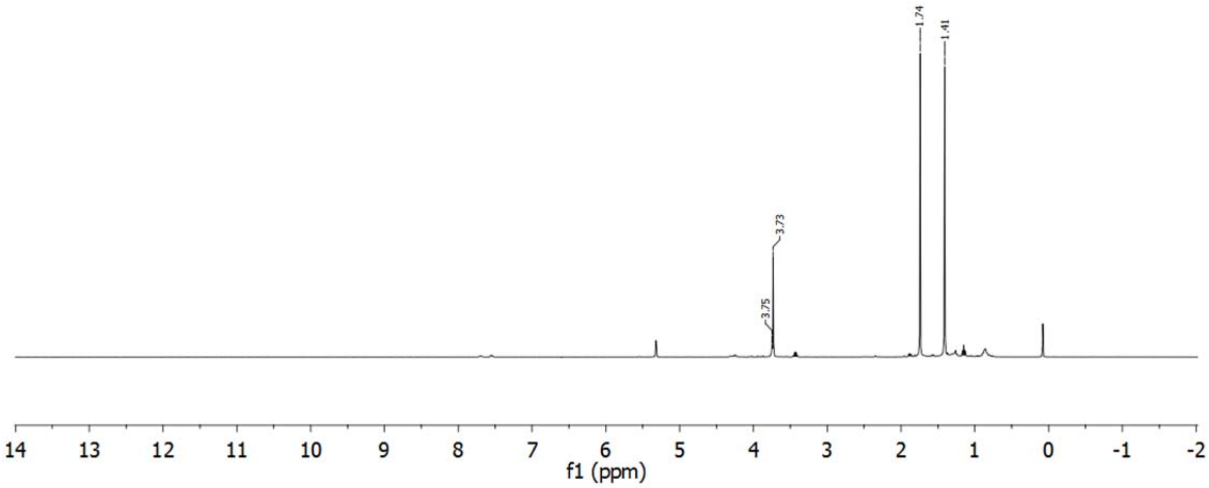

Figure S12. ^{1}H NMR spectrum of [RhCl(**6'**)(CO)$_2$] (CD_2Cl_2, 400 MHz). The signal at 0.1 ppm is due to silicon grease. The minor signals 7.5, 3.4, 1.3 and 0.8 ppm are due to unknown impurities.

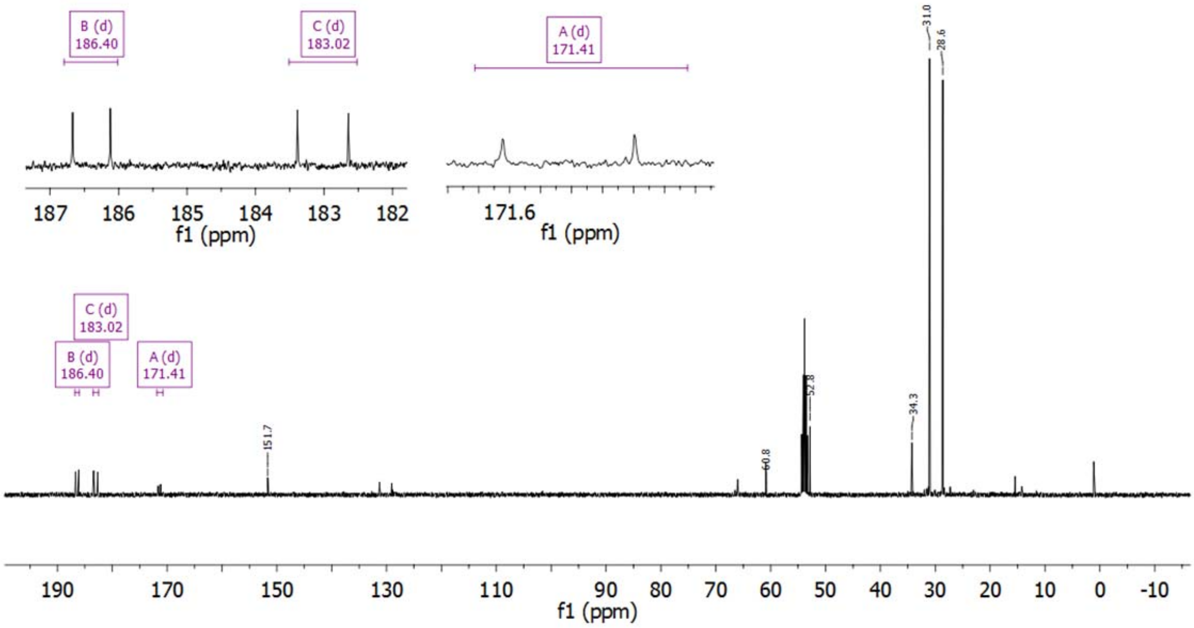

Figure S13. ^{13}C NMR spectrum of [RhCl(**6’)**(CO)$_2$] (CD_2Cl_2, 100 MHz). The signal at 1 ppm is due to silicon grease. The minor signals at 133, 128, 65 and 15 ppm are due to unknown impurities.

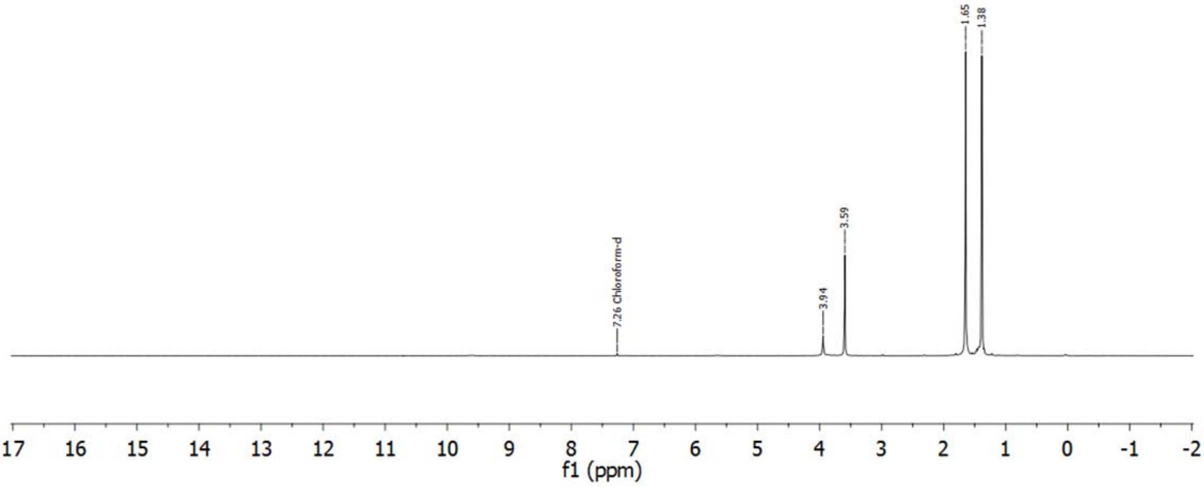

Figure S14. ^{1}H NMR spectrum of [CuCl(**6’**)] ($CDCl_3$, 400 MHz).

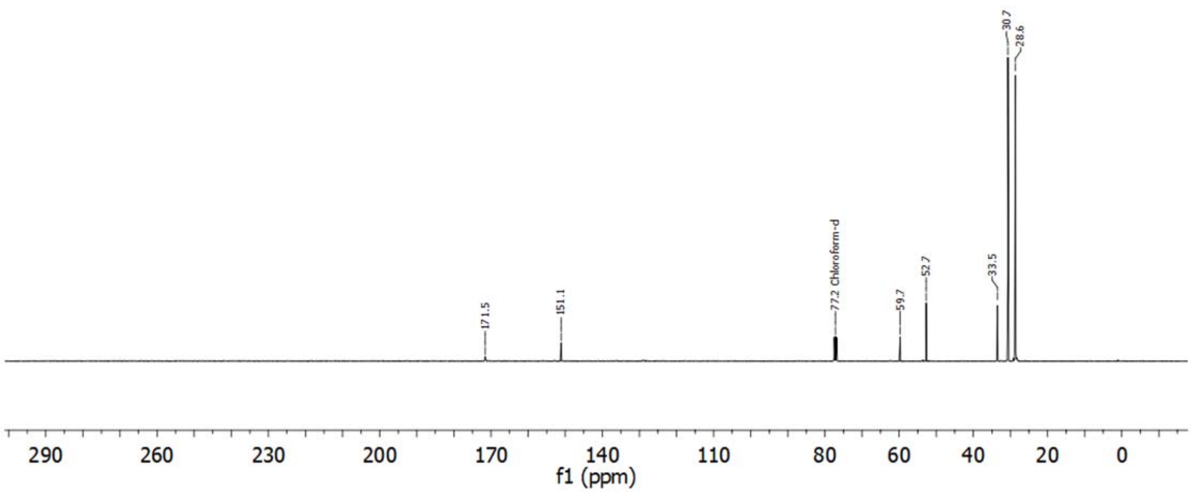

Figure S15. ^{13}C NMR spectrum of [CuCl(**6’**)] ($CDCl_3$, 100 MHz).

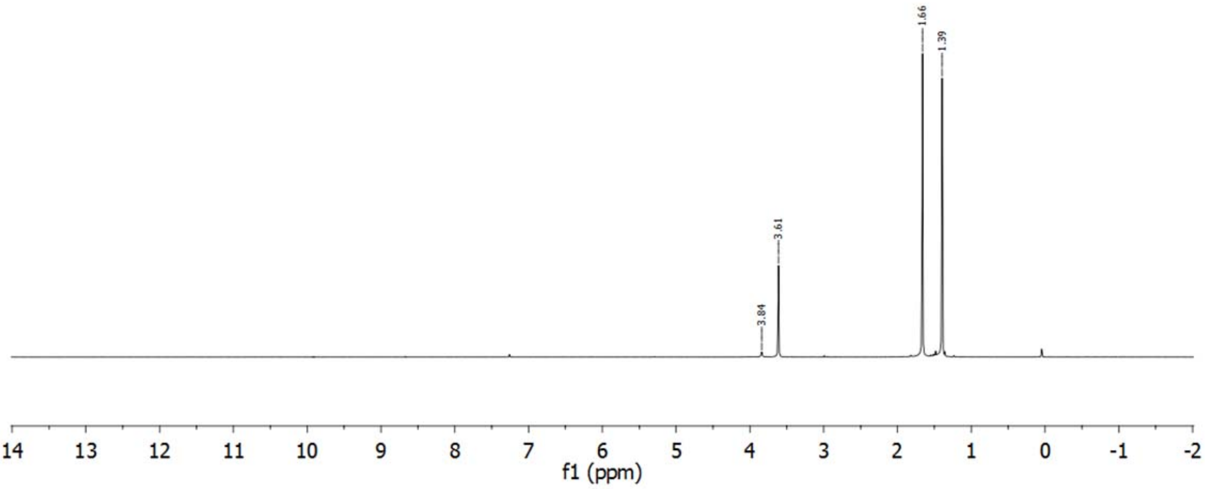

Figure S16. ^{1}H NMR spectrum of [CuBr(**6’**)] ($CDCl_3$, 400 MHz). The signal at 0.1 ppm is due to silicon grease.

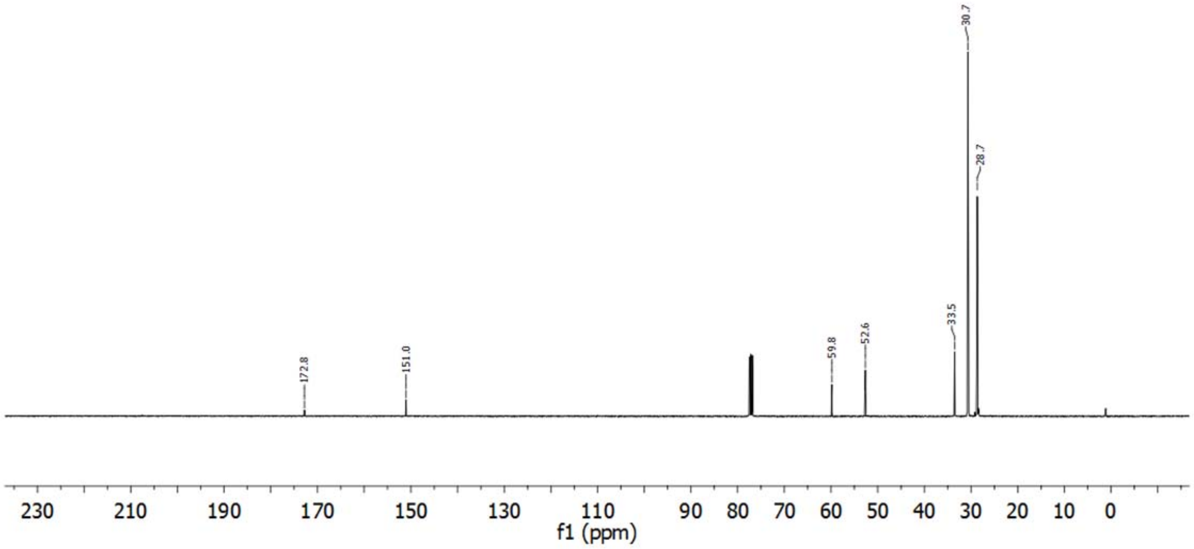

Figure S17. ^{13}C NMR spectrum of [CuBr(**6'**)] ($CDCl_3$, 100 MHz). The signal at 1 ppm is due to silicon grease.

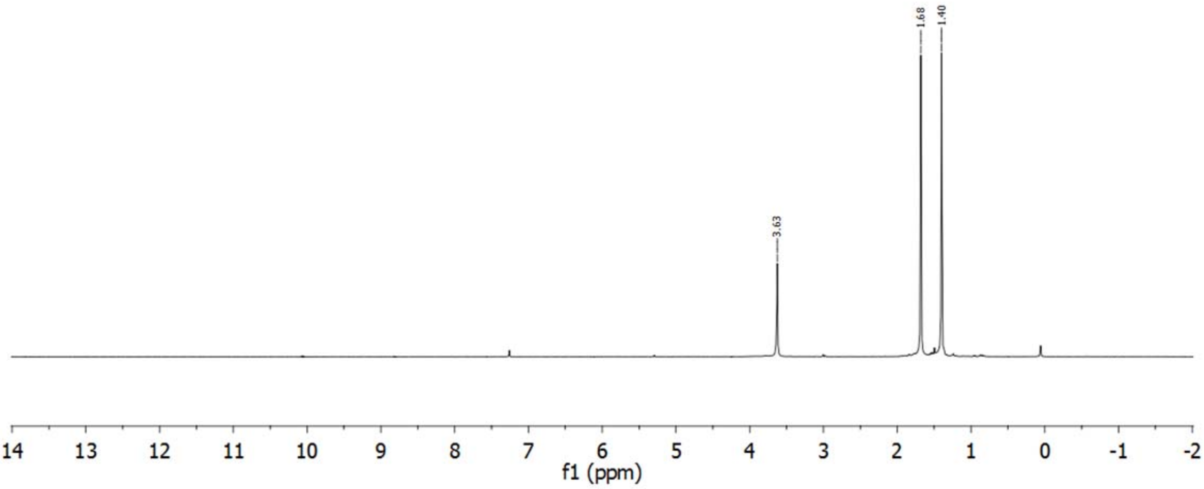

Figure S18. ^{1}H NMR spectrum of [CuI(**6'**)] ($CDCl_3$, 400 MHz). The signal at 0.1 ppm is due to silicon grease.

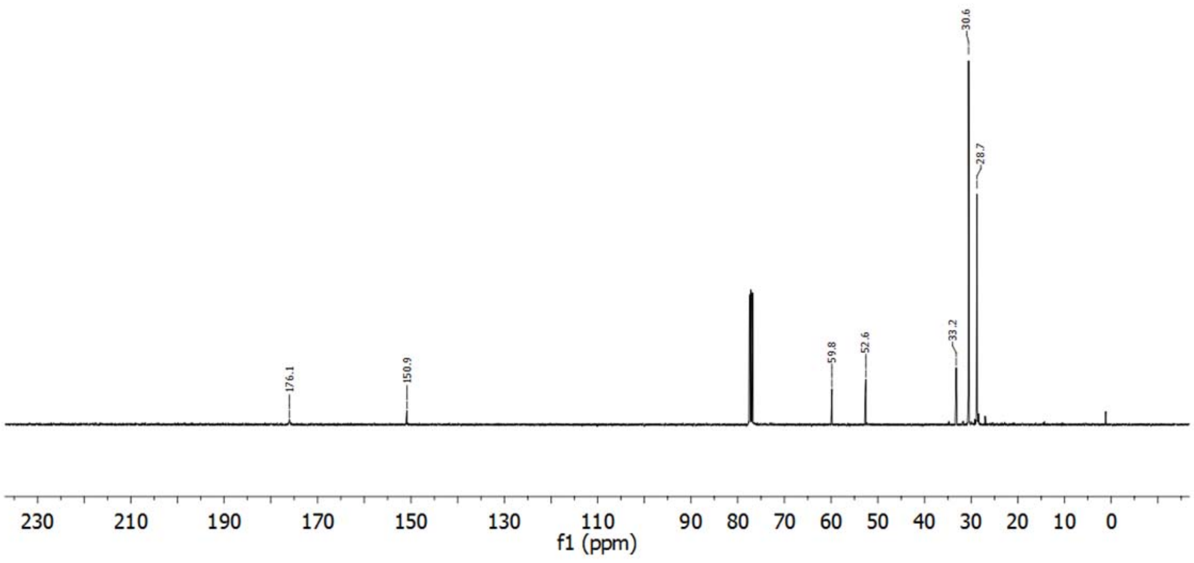

Figure S19. ^{13}C NMR spectrum of [CuI(**6’**)] ($CDCl_3$, 100 MHz). The signal at 1 ppm is due to silicon grease.

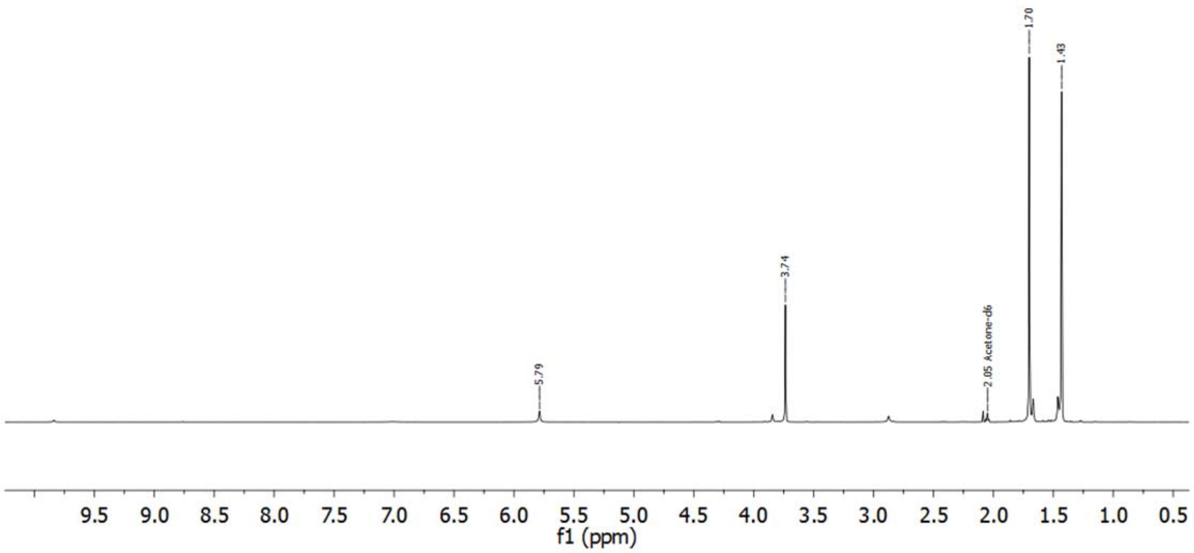

Figure S20. ^{1}H NMR spectrum of [AgCl(**6’**)] (acetone-d_6, 400 MHz). The signal at 2.85 ppm is due to water. The minor signals at 3.84, 1.67 and 1.46 ppm are due to **6**H^+. The signal at 2.07 ppm is due to an unknown impurity.

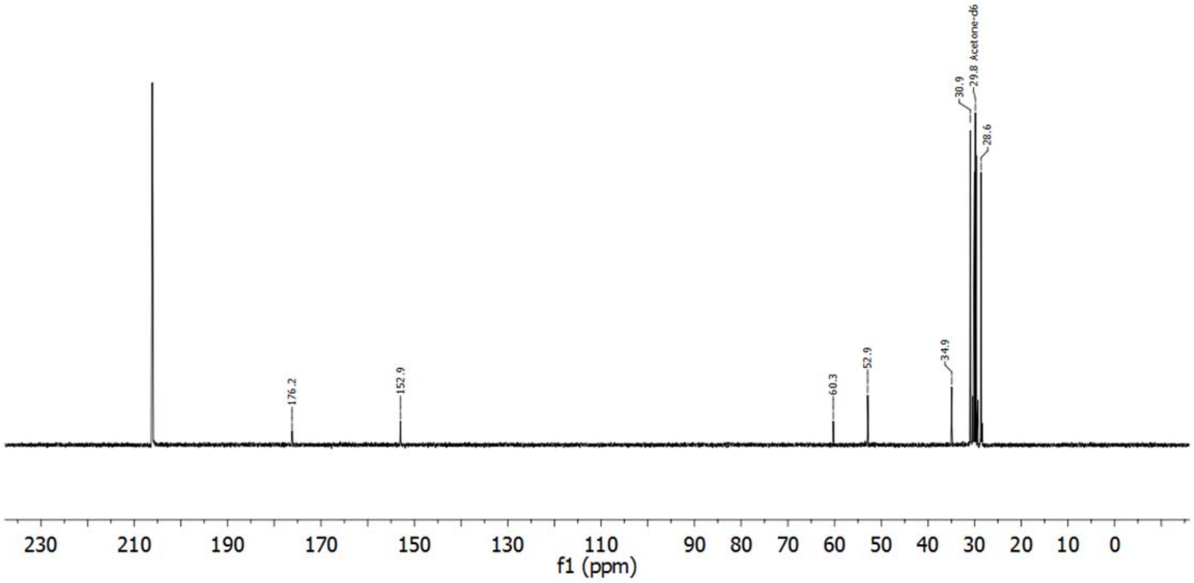

Figure S21. ^{13}C NMR spectrum of [AgCl(**6'**)] (acetone-d_6, 100 MHz).

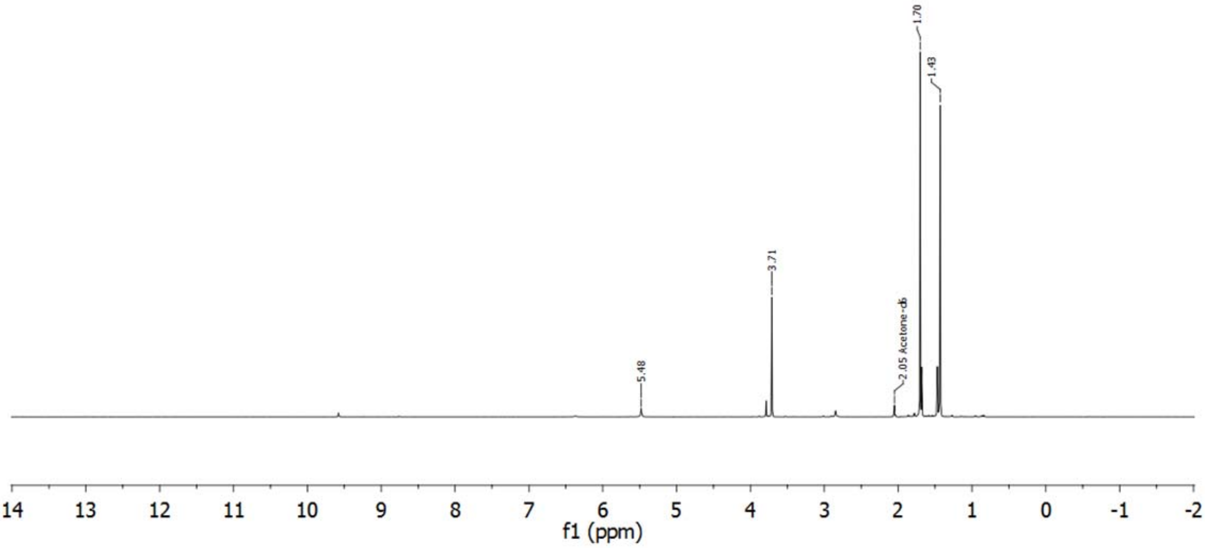

Figure S22. ^{1}H NMR spectrum of [AgBr(**6'**)] (acetone-d_6, 400 MHz). The signal at 2.85 ppm is due to water. The minor signals at 3.80, 1.68 and 1.47 ppm are due to **6**H$^+$.

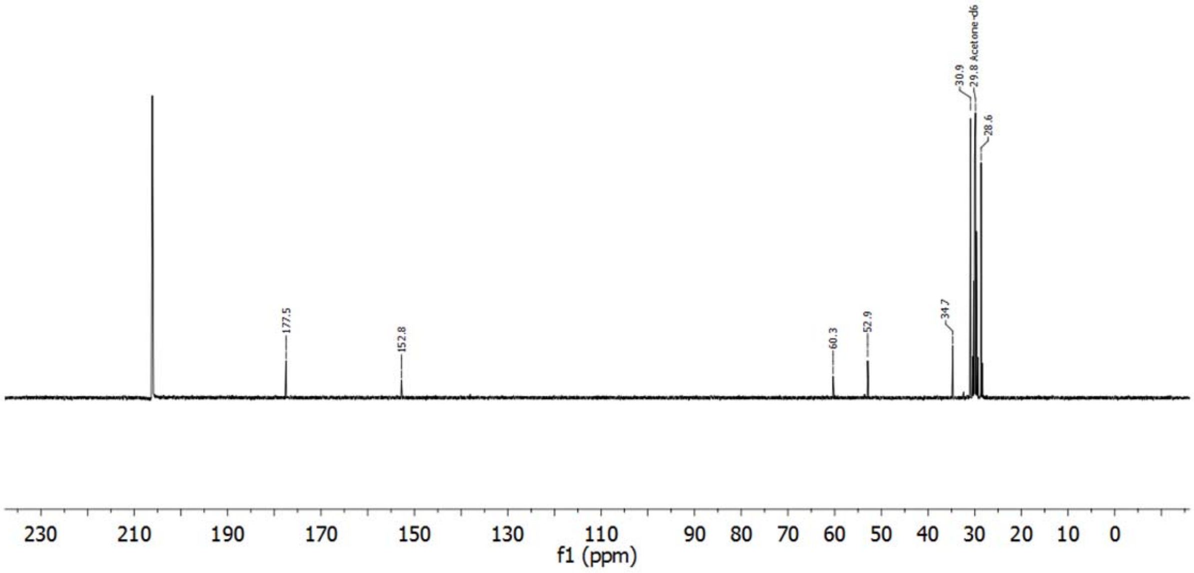

Figure S23. ^{13}C NMR spectrum of [AgBr(**6'**)] (acetone-d_6, 100 MHz).

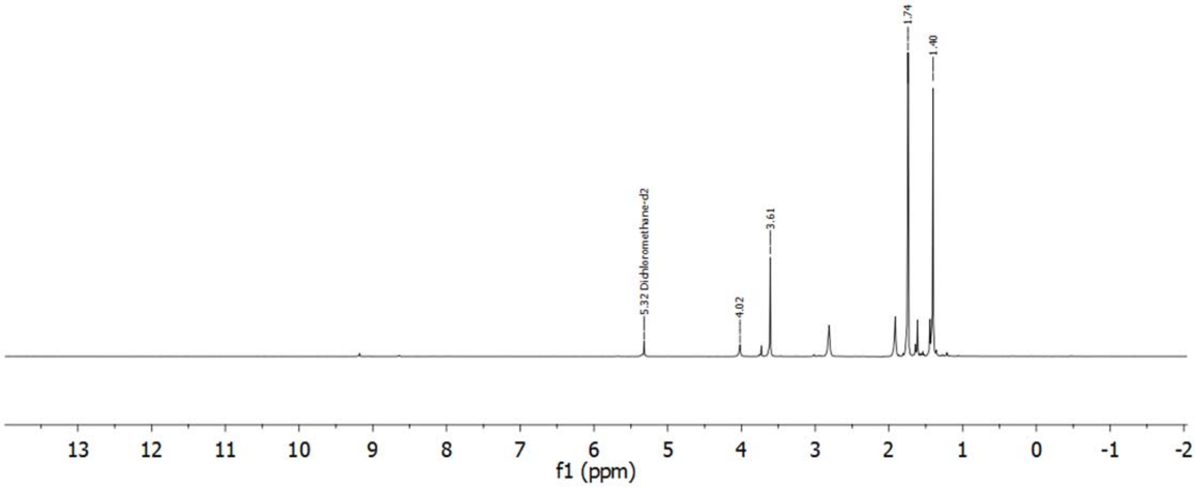

Figure S24. ^{1}H NMR spectrum of [AuCl(**6'**)] (CD_2Cl_2, 500 MHz). The signals at 2.81 ppm and 1.91 ppm are due to residual tetrahydrothiophene. The minor signals at 3.73, 1.61 and 1.44 ppm are due to **6**H^+.

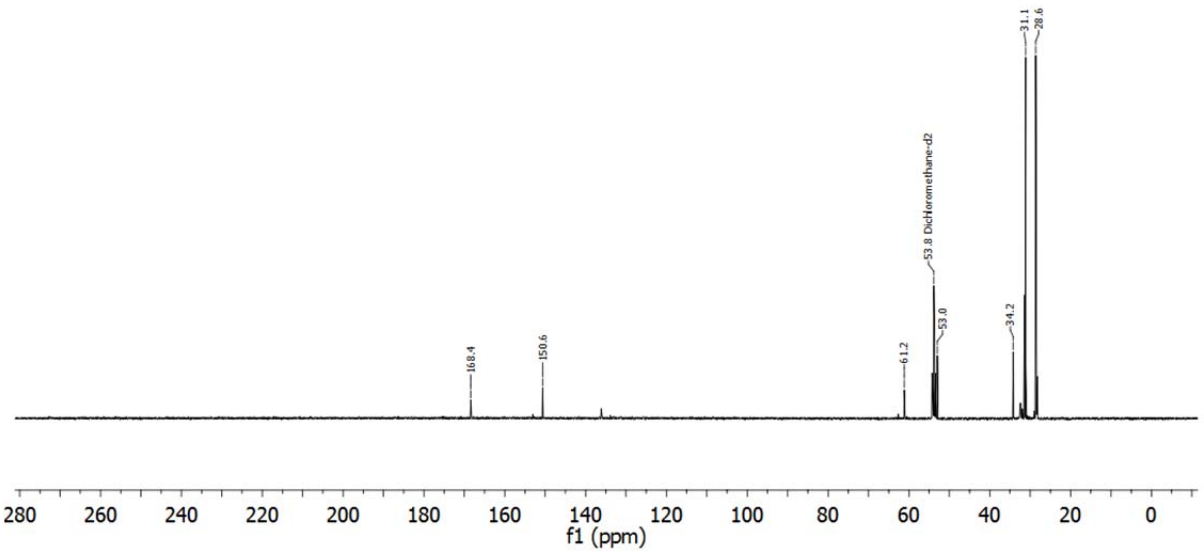

Figure S25. ^{13}C NMR spectrum of [AuCl(**6’**)] (CD_2Cl_2, 125 MHz). The signal at 31.4 ppm is due to tetrahydrothiophene. The signals at 153.0, 136.1, 62.6, 32.4, 28.4 and 28.2 ppm are due to **6**H^+.

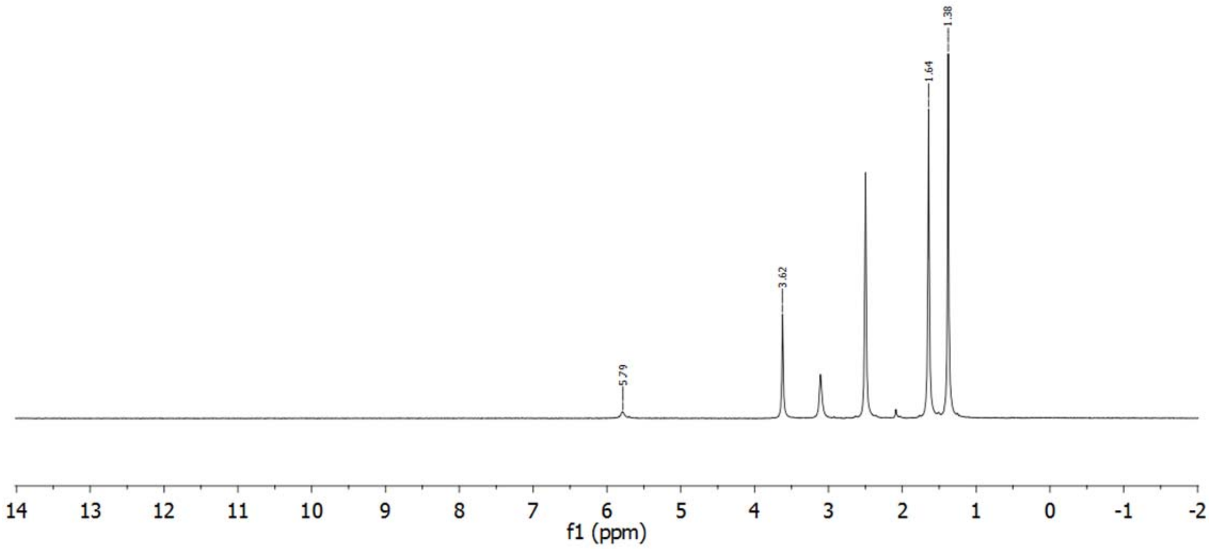

Figure S26. ^{1}H NMR spectrum of [CuCl(**6’**)$_2$] (DMSO-d_6, 500 MHz, 70 °C). The signals at 3.10 and 2.05 ppm are due to water and acetone, respectively.

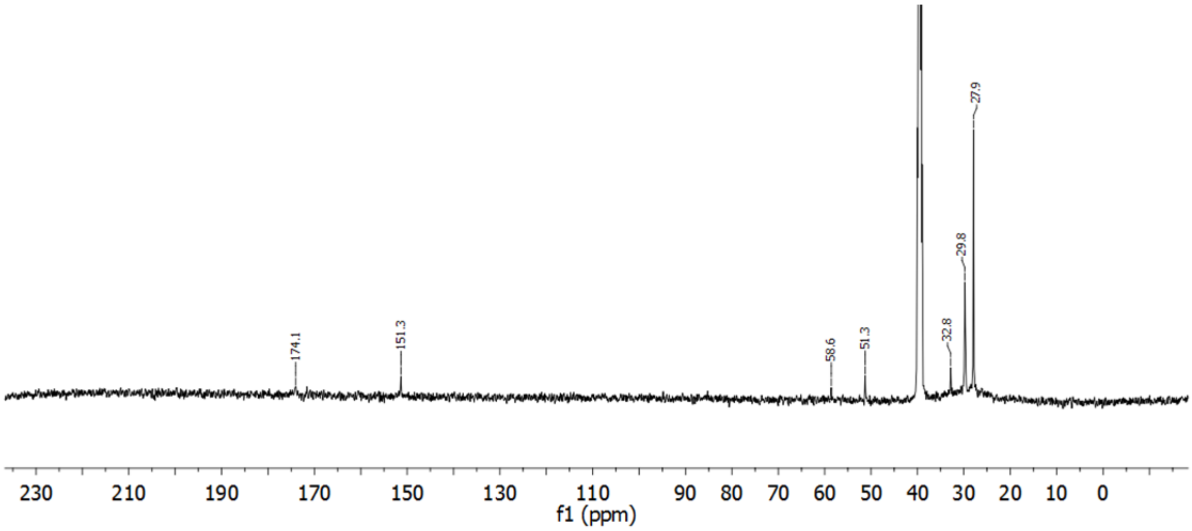

Figure S27. ^{13}C NMR spectrum of [CuCl(**6'**)$_2$] (DMSO-d_6, 125 MHz, 70 °C).

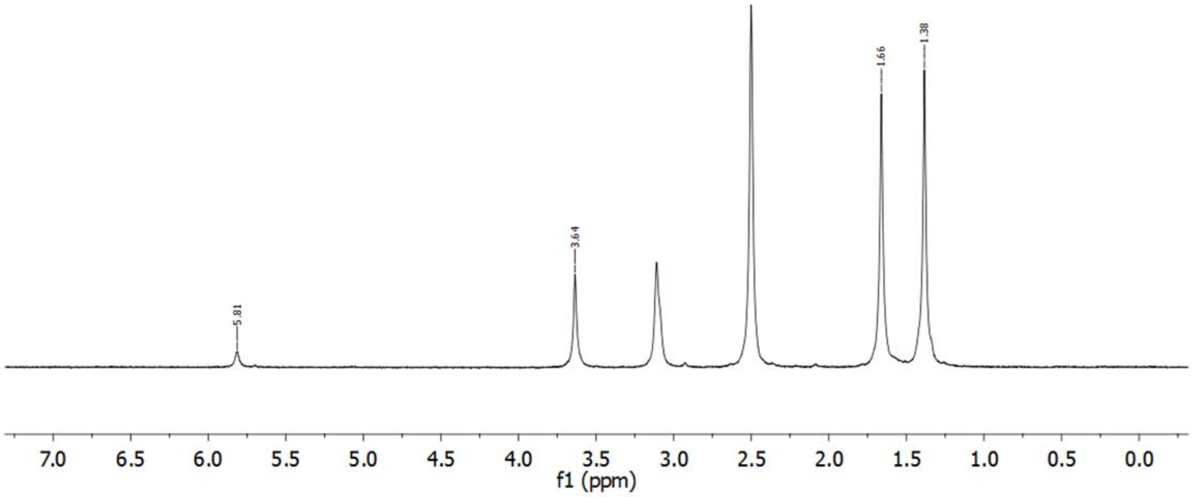

Figure S28. ^{1}H NMR spectrum of [CuBr(**6'**)$_2$] (DMSO-d_6, 500 MHz, 70 °C). The signal at 3.10 ppm is due to water.

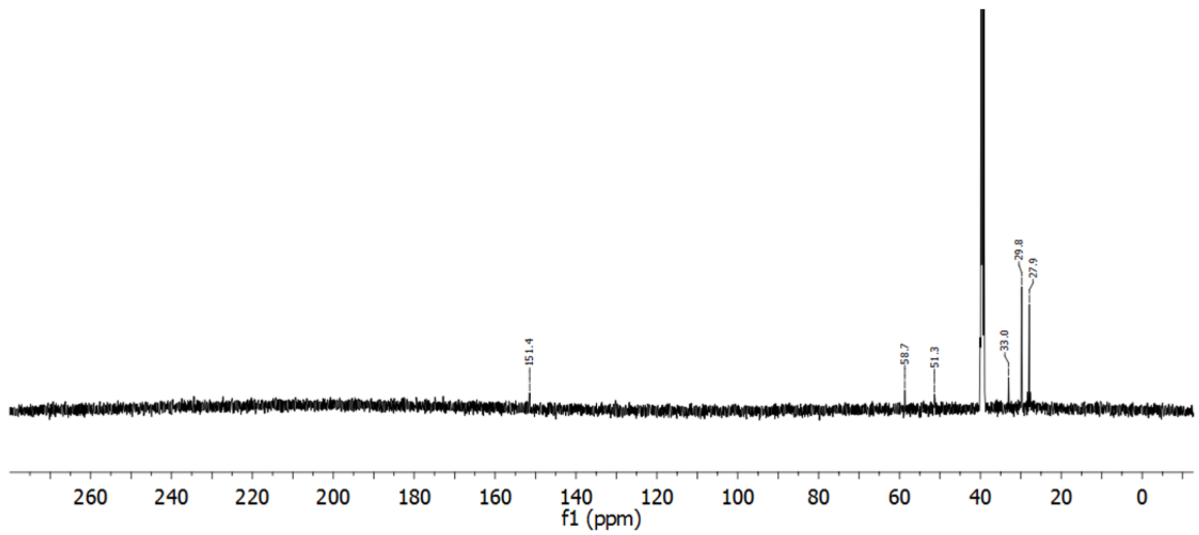

Figure S29. ^{13}C NMR spectrum of [CuBr(**6’**)$_2$] (DMSO-d_6, 125 MHz, 70 °C).

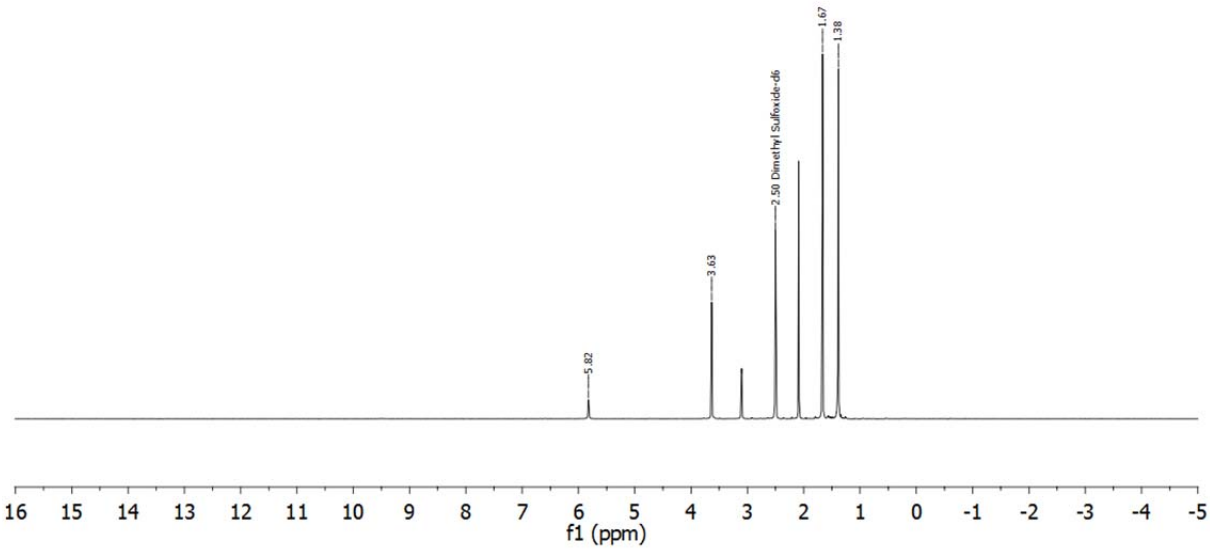

Figure S30. ^{1}H NMR spectrum of [CuI(**6’**)$_2$] (DMSO-d_6, 500 MHz, 70 °C). The signals at 3.10 and 2.09 ppm are due to water and acetone, respectively.

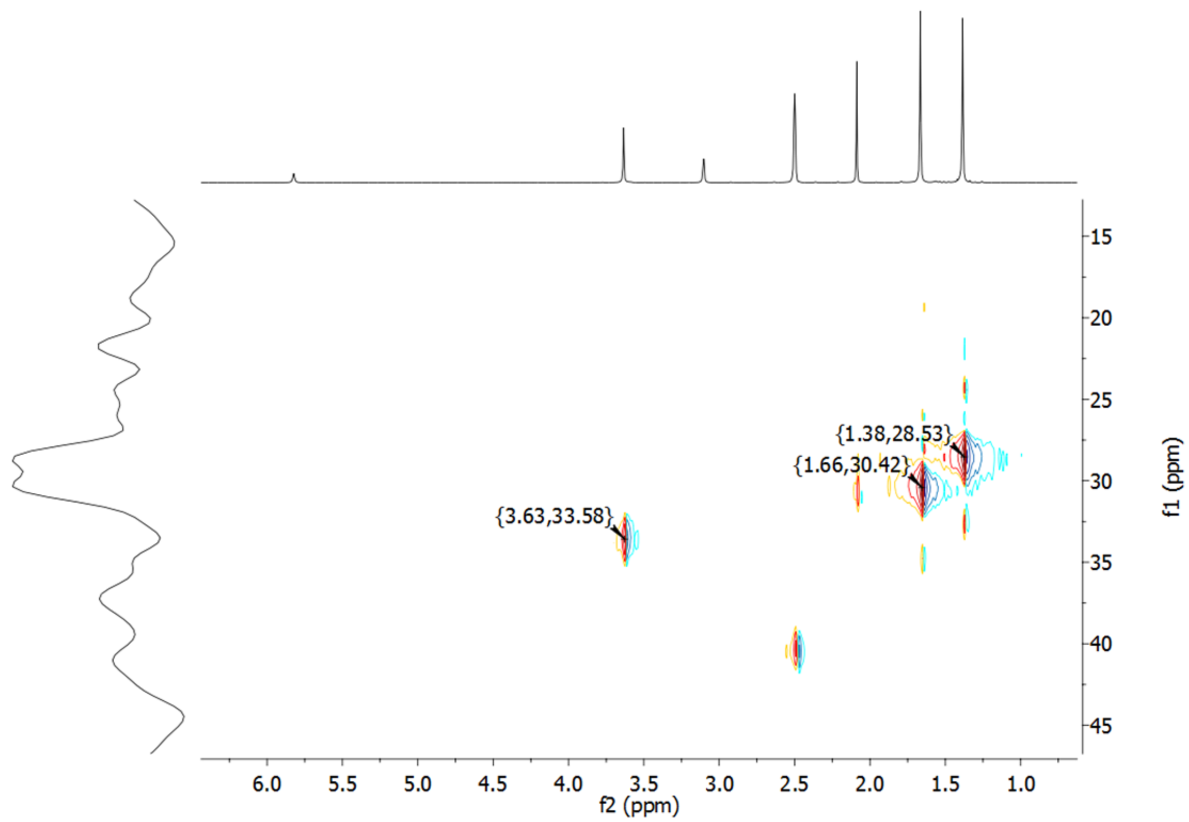

Figure S31. HSQC NMR spectrum of [CuI(**6’**)$_2$] (DMSO-d_6, 500 MHz, 70 °C).

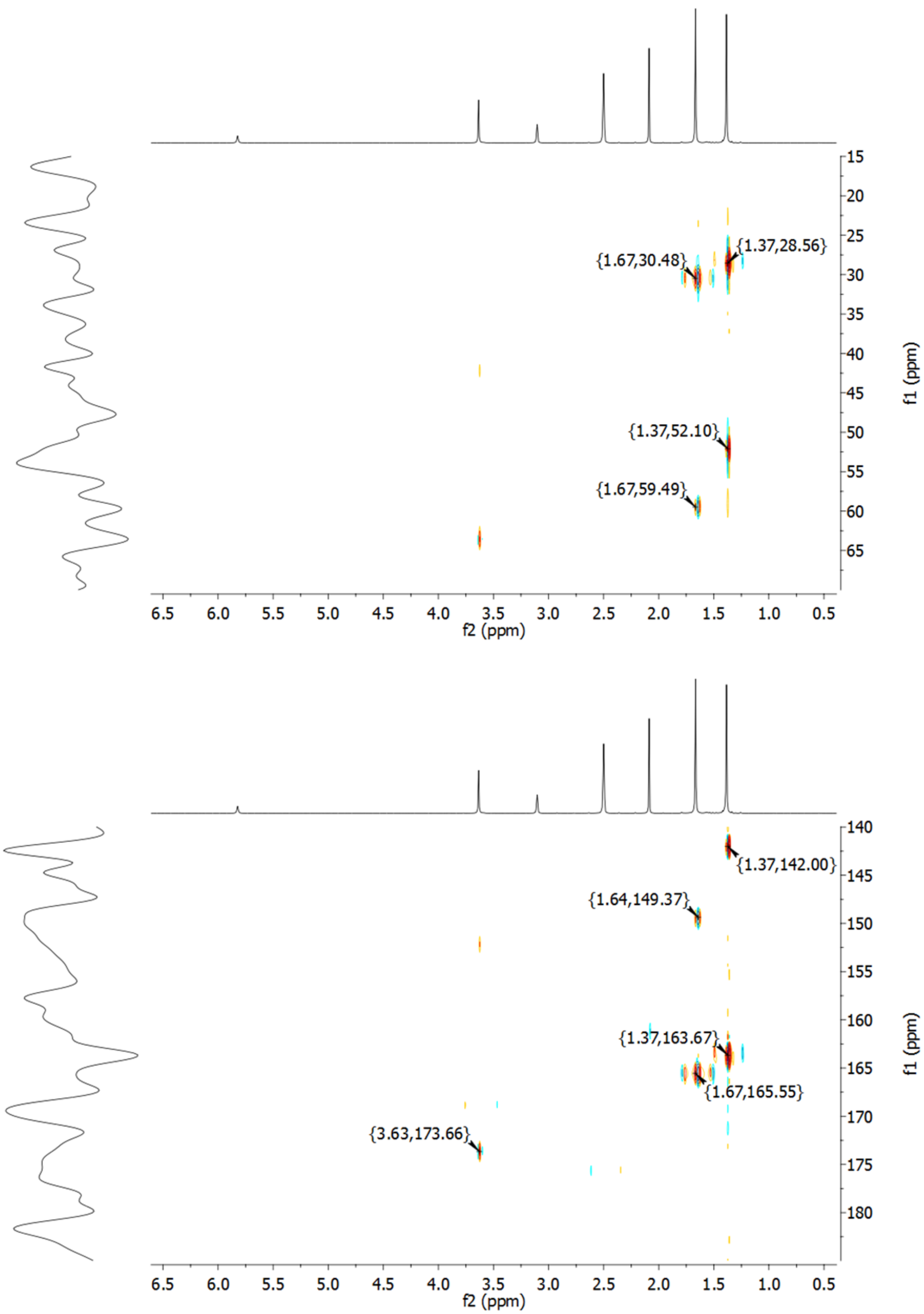

Figure S32. HMBC NMR spectrum of [CuI(**6'**)$_2$] (DMSO-d_6, 500 MHz, 70 °C), displayed in two sections.

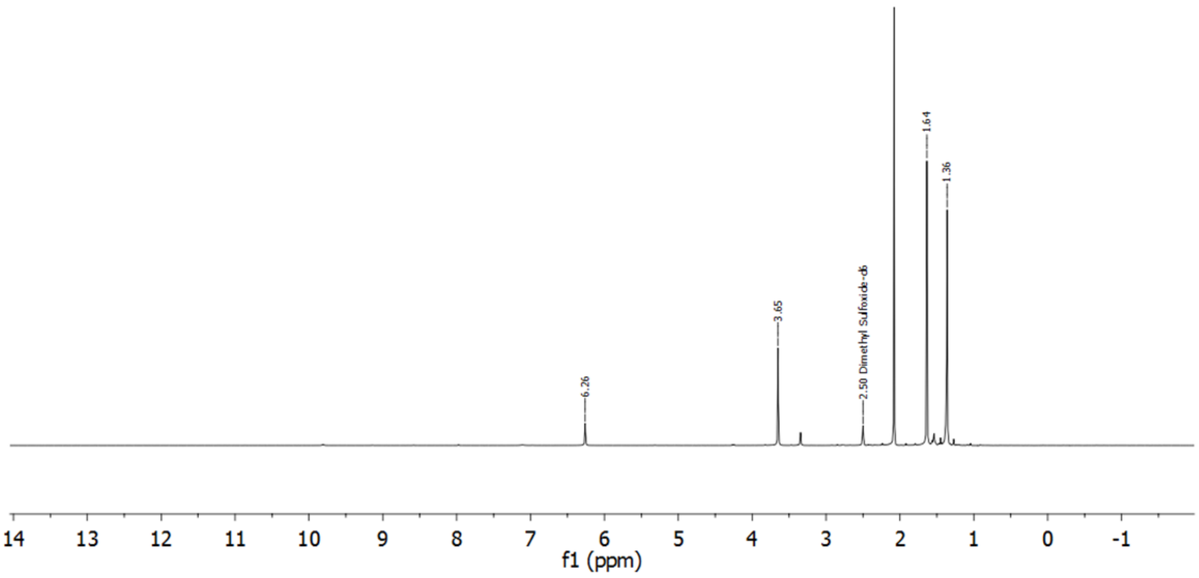

Figure S33. ^{1}H NMR spectrum of [Ag(**6'**)$_2$]Cl (DMSO-d_6, 400 MHz). The signals at 3.33 ppm and 2.10 ppm are due to water and acetone, respectively.

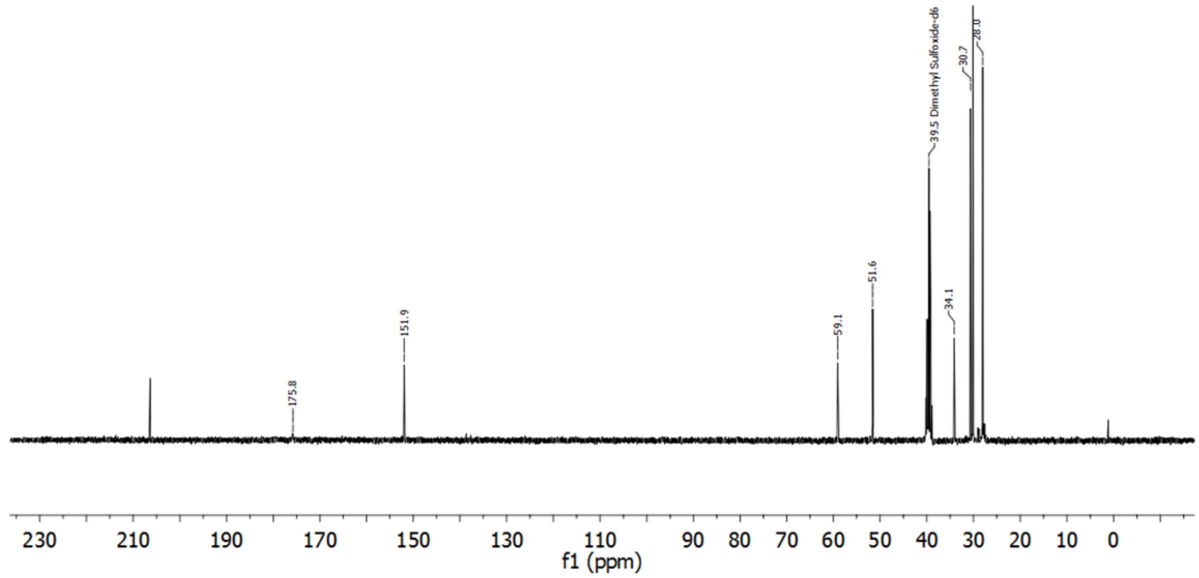

Figure S34. ^{13}C NMR spectrum of [Ag(**6'**)$_2$]Cl (DMSO-d$_6$, 100 MHz). The signals at 205.9 ppm and 30.6 ppm are due to acetone.

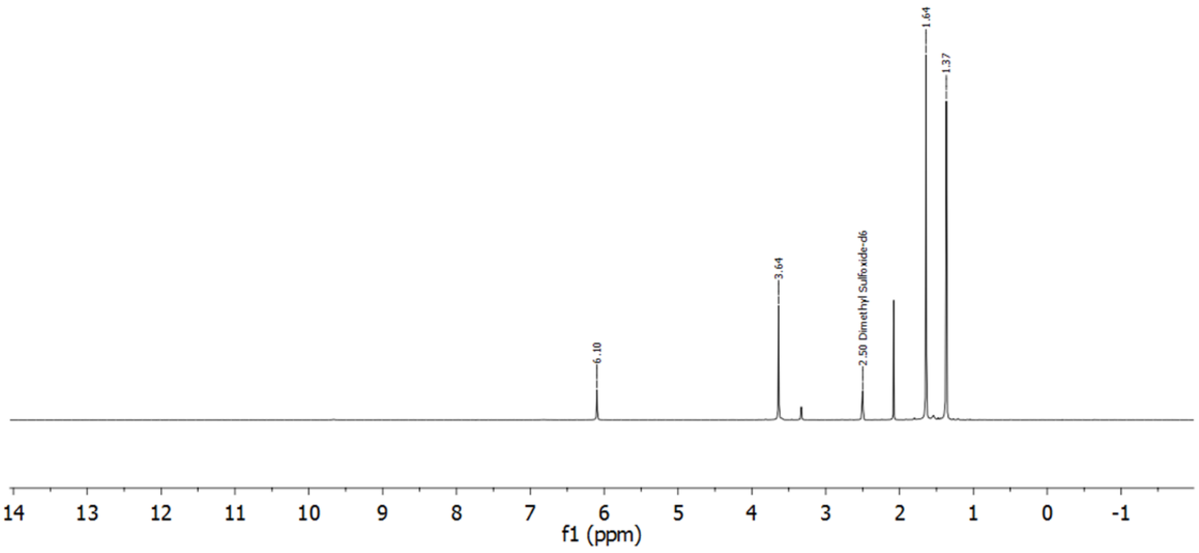

Figure S35. ^{1}H NMR spectrum of [Ag(**6’**)$_2$]Br (DMSO-d_6, 400 MHz). The signals at 3.33 ppm and 2.10 ppm are due to water and acetone, respectively.

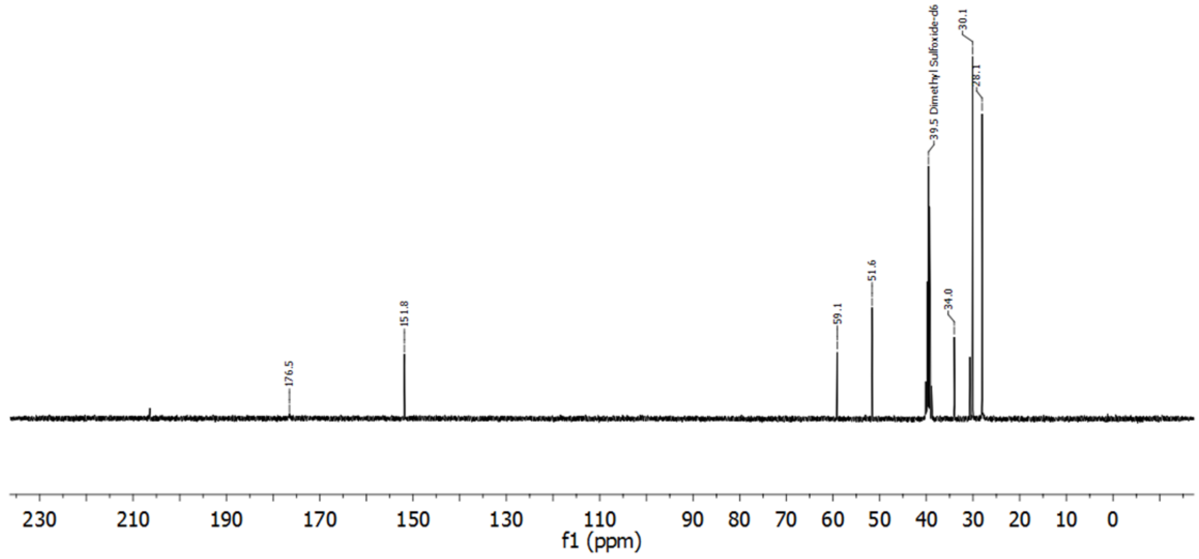

Figure S36. ^{13}C NMR spectrum of [Ag(**6’**)$_2$]Br (DMSO-d_6, 100 MHz). The signals at 205.9 ppm and 30.6 ppm are due to acetone.

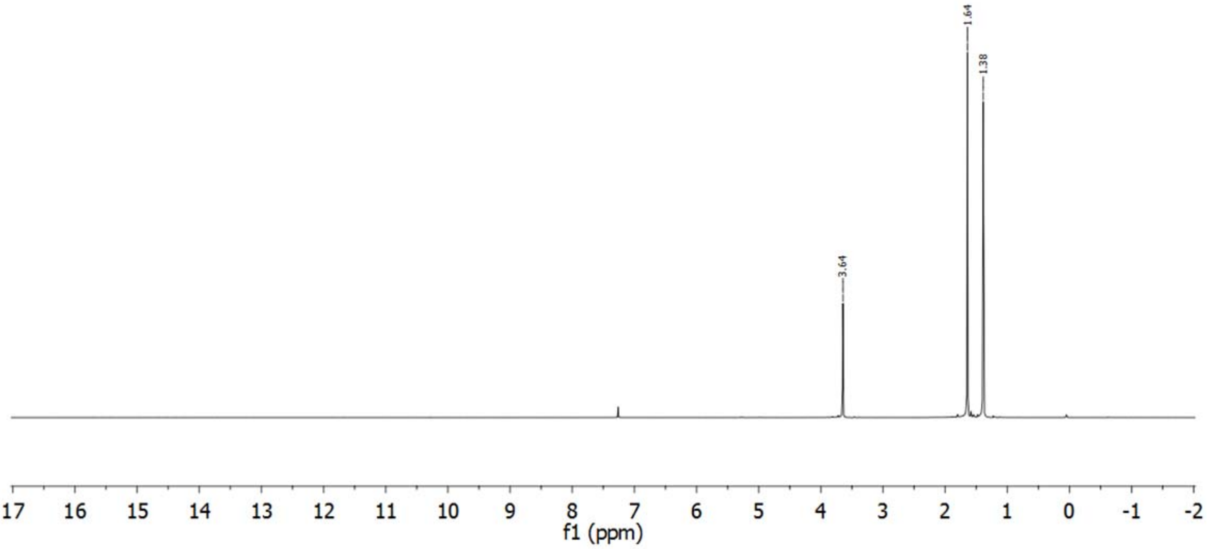

Figure S37. ^{1}H NMR spectrum of [Ag(**6'**)$_2$](OTf) ($CDCl_3$, 400 MHz).

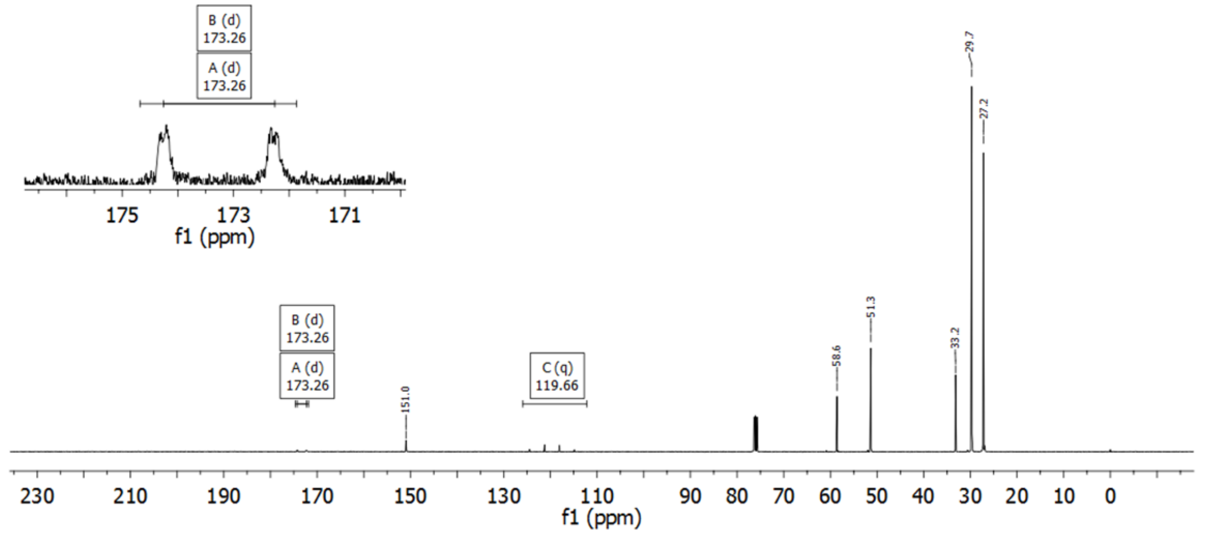

Figure S38. ^{13}C NMR spectrum of [Ag(**6'**)$_2$](OTf) ($CDCl_3$, 100 MHz).

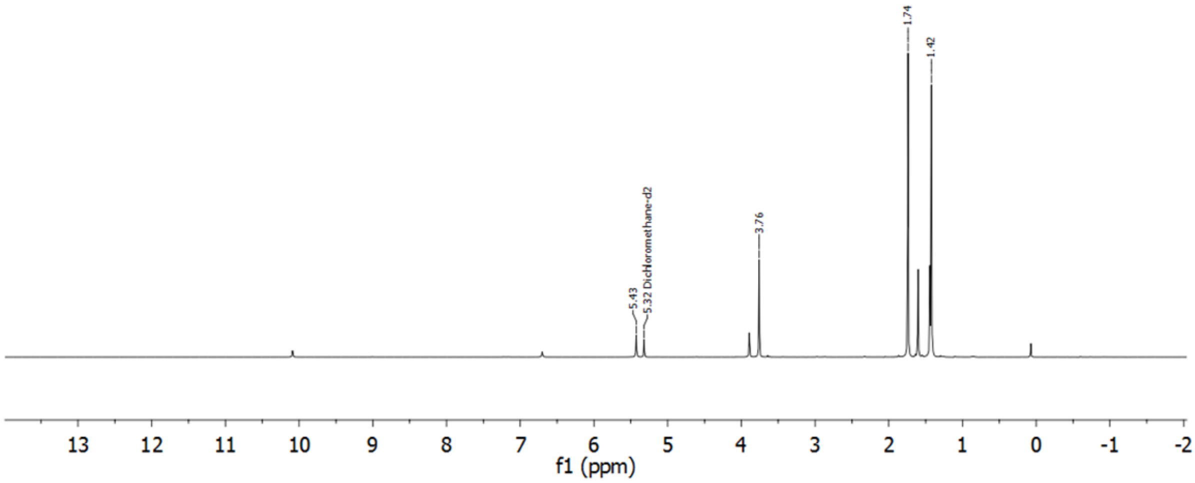

Figure S39. ^{1}H NMR spectrum of [Au(**6'**)$_2$]Cl (CD_2Cl_2, 500 MHz). The signal at 0.1 ppm is due to silicon grease. The minor signals at 10.09, 6.70, 3.89, 1.60 and 1.44 ppm are due to **6**H^+.

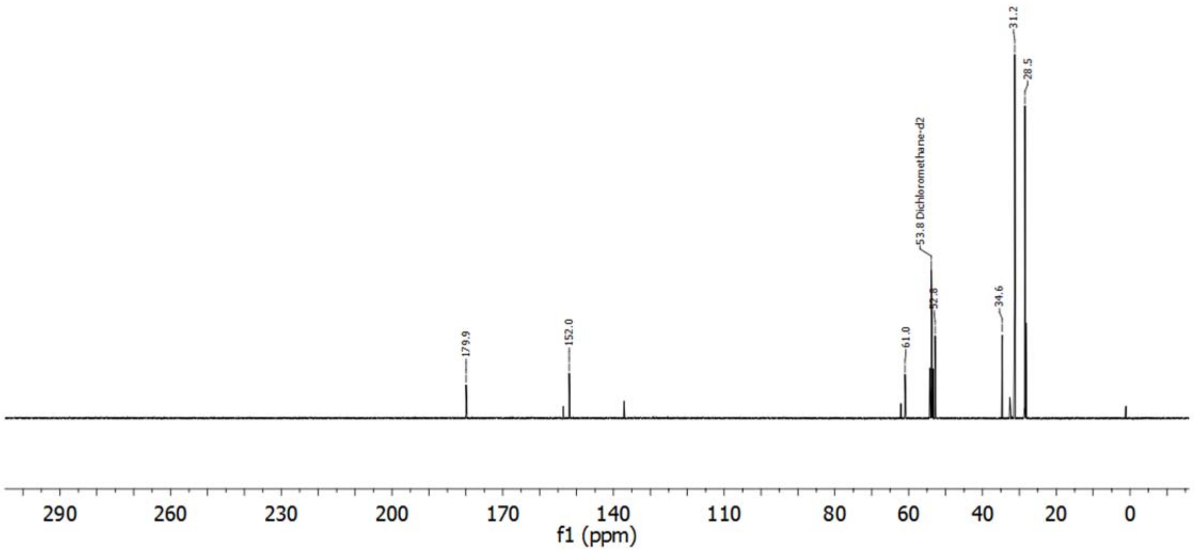

Figure S40. ^{13}C NMR spectrum of [Au(**6'**)$_2$]Cl (CD_2Cl_2, 125 MHz). The signal at 1 ppm is due to silicon grease. The minor signals at 153.6, 137.1, 62.1, 53.4, 32.6, 28.5 and 28.2 ppm are due to **6**H^+.
